# Reduced Neurosteroid Exposure Following Preterm Birth and Its’ Contribution to Neurological Impairment: A Novel Avenue for Preventative Therapies

**DOI:** 10.3389/fphys.2019.00599

**Published:** 2019-05-15

**Authors:** Julia C. Shaw, Mary J. Berry, Rebecca M. Dyson, Gabrielle K. Crombie, Jonathan J. Hirst, Hannah K. Palliser

**Affiliations:** ^1^School of Biomedical Sciences and Pharmacy, University of Newcastle, Newcastle, NSW, Australia; ^2^Mothers and Babies Research Centre, Hunter Medical Research Institute, University of Newcastle, Newcastle, NSW, Australia; ^3^Department of Paediatrics and Child Health, University of Otago, Wellington, Wellington, New Zealand; ^4^Centre for Translational Physiology, University of Otago, Wellington, Wellington, New Zealand

**Keywords:** neurosteroid, preterm birth, GABAA receptor (GABAAR), myelin, ganaxolone

## Abstract

Children born preterm are at an increased risk of developing cognitive problems and neuro-behavioral disorders such as attention deficit hyperactivity disorder (ADHD) and anxiety. Whilst neonates born at all gestational ages, even at term, can experience poor cognitive outcomes due to birth-complications such as birth asphyxia, it is becoming widely known that children born preterm in particular are at significant risk for learning difficulties with an increased utilization of special education resources, when compared to their healthy term-born peers. Additionally, those born preterm have evidence of altered cerebral myelination with reductions in white matter volumes of the frontal cortex, hippocampus and cerebellum evident on magnetic resonance imaging (MRI). This disruption to myelination may underlie some of the pathophysiology of preterm-associated brain injury. Compared to a fetus of the same post-conceptional age, the preterm newborn loses access to *in utero* factors that support and promote healthy brain development. Furthermore, the preterm *ex utero* environment is hostile to the developing brain with a myriad of environmental, biochemical and excitotoxic stressors. Allopregnanolone is a key neuroprotective fetal neurosteroid which has promyelinating effects in the developing brain. Preterm birth leads to an abrupt loss of the protective effects of allopregnanolone, with a dramatic drop in allopregnanolone concentrations in the preterm neonatal brain compared to the fetal brain. This occurs in conjunction with reduced myelination of the hippocampus, subcortical white matter and cerebellum; thus, damage to neurons, astrocytes and especially oligodendrocytes of the developing nervous system can occur in the vulnerable developmental window prior to term as a consequence reduced allopregnanolone. In an effort to prevent preterm-associated brain injury a number of therapies have been considered, but to date, other than antenatal magnesium sulfate and corticosteroid therapy, none have become part of standard clinical care for vulnerable infants. Therefore, there remains an urgent need for improved therapeutic options to prevent brain injury in preterm neonates. The actions of the placentally derived neurosteroid allopregnanolone on GABA_A_ receptor signaling has a major role in late gestation neurodevelopment. The early loss of this intrauterine neurotrophic support following preterm birth may be pivotal to development of neurodevelopmental morbidity. Thus, restoring the *in utero* neurosteroid environment for preterm neonates may represent a new and clinically feasible treatment option for promoting better trajectories of myelination and brain development, and therefore reducing neurodevelopmental disorders in children born preterm.

## Introduction

Preterm birth is the leading cause of death and neurodevelopmental related disability in early life (Goldenberg et al., 2008). In resource rich nations such as Australia, the incidence of moderate-late preterm birth specifically now accounts for ∼80% of all preterm births (Cheong and Doyle, 2012; Frey and Klebanoff, 2016). These neonates have a high survival rate and a low incidence of gross neuroanatomical damage on routine clinical imaging; however, there is increasing evidence of microcystic white matter injury when assessed using MRI. Even amongst those infants who appear well at the time of hospital discharge, and are free of gross neuroanatomical lesions, there remains a high burden of later cognitive difficulties and neurodevelopmental disorders such as anxiety and attention deficit hyperactivity disorder (ADHD) (Ananth and Vintzileos, 2006; Chyi et al., 2008; Moster et al., 2008; Petrini et al., 2009; Loe et al., 2011; Baron et al., 2012; Cheong and Doyle, 2012; Potijk et al., 2012). The long-term individual, familial and socio-economic burden of these issues remains profound; with rates of preterm birth at around 10%, and with increasing numbers of children surviving, there is an urgent need to develop novel therapeutic options to mitigate, or prevent, the ongoing neurological burden of preterm birth.

Myelination of white matter tracts continues throughout late gestation and following birth in areas such as the hippocampus and cerebellum: reduction in brain volumes and functionality of these regions are evident in children that were born preterm (Rivkin, 1997; Rees and Inder, 2005; Rees et al., 2008; Volpe, 2008). In particular myelination by mature oligodendrocytes is ongoing throughout this late gestation stage and is vulnerable to insults and excitotoxic damage associated with early exposure to the *ex utero* environment (Arnold and Trojanowski, 1996; Back et al., 2002; Matsusue et al., 2014). *In utero*, the fetal neurosteroid allopregnanolone is responsible for protection from neurological insults, modulating fetal behavior leading to the onset of a ‘sleep-like state,’ and promoting myelination through its action on the inhibitory GABA_A_ receptors of the central nervous system (CNS) (Nicol et al., 1998; Nguyen et al., 2003; Herd et al., 2007). Importantly, recent studies suggest that behavioral and cognitive outcomes are tightly linked with gestational age (Berry et al., 2018). Any decrement in gestation, even across ‘early term’ (37/38 weeks’ gestation) is associated with, on a population basis, impaired cognitive and developmental outcomes compared to outcomes found in children born at full term (39–40 weeks gestational age) (Berry et al., 2018).

Birth is necessarily associated with the loss of the fetus from the placenta-maternal unit, and therefore separation from any trophic factors derived from either mother or placenta. Preterm birth results in the premature loss of placentally supplied allopregnanolone during a period when it is critical for optimal neurodevelopment (Kelleher et al., 2013). Whilst neurosteroid therapy has been evaluated for the treatment of traumatic brain injury (TBI) and epilepsy (Nohria and Giller, 2007; Wright et al., 2007; Xiao et al., 2008; Reddy and Rogawski, 2012), therapeutic use of neurosteroids following preterm birth requires further evaluation.

## Neurological Outcomes of Preterm Birth

Despite only comprising 10% of births, preterm birth is the leading cause of death and neurodevelopmental related disability in neonates, accounting for up to 50% of neonatal deaths (Simmons et al., 2010). Furthermore, the ongoing morbidity risks of preterm birth remain unacceptably high with up to 50% of survivors developing some form of long-term neurodevelopmental disability (Mathews et al., 2002; Ananth and Vintzileos, 2006). Cerebral white matter injury in the preterm infant varies based on gestational age at the time of birth. Historically, injury following early preterm birth was characterized by intraventricular hemorrhage and, or, periventricular leukomalacia (PVL) (Volpe, 2001, 2009). In survivors of early preterm birth weighing <1,500 g approximately 10% develop cerebral palsy as a result of these gross insults and necrosis (Volpe, 2003). However, with improvements in perinatal care these gross structural lesions are now far less common, whereas diffuse white matter injury (DWMI) demonstrable on MRI, but not routine screening cranial ultrasound, is increasingly recognized as the key contributor to the pathophysiology of preterm-associated brain injury. It is now established that impaired cognition, sensory and psychological functioning in children born preterm is associated with DWMI (van Tilborg et al., 2016). Furthermore, DWMI is a recognized risk factor for the development of neurobehavioral disorders such as autism-spectrum disorders and ADHD (van Tilborg et al., 2016). The underlying pathophysiological mechanisms of DWMI are poorly understood but are suggested to be due to immature oligodendrocyte arrest resulting in impaired myelination.

In infants that were born <32 weeks’ gestation, it has recently been shown that reductions in white matter volume in areas such as the fornix and the cingulum observed by MRI at the time of birth remained present until 19 years of age and were associated with impairments in memory functions (Caldinelli et al., 2017). The Stockholm Neonatal Project has also recently published the results of a longitudinal trial following infants born <36 weeks’ gestation up until 18 years of age when they undertook psychological assessment including general intelligence and executive functioning measures. Significantly poorer outcomes were observed for preterm children in areas such as IQ, attention, working memory, and cognitive flexibility (Vollmer et al., 2017). Most importantly, however, is that the executive functioning deficits did not correlate with reductions in white matter or gray matter volumes evident by MRI following birth, but the microstructure of white matter tracts was altered at adolescence. Thus, this study found that following preterm birth, and in the absence of obvious perinatal brain injury, the alterations observed in white matter microstructure during adolescence correlate with executive function and general cognitive abilities. Furthermore, it suggested that disruption to neural pathways, as opposed to reductions in brain volume, is involved in the impairment of neurodevelopment following preterm birth. In addition to established preterm birth related disorders, such as cerebral palsy, there is now a growing body of evidence suggesting that preterm infants from moderate-late preterm pregnancies are much more likely to develop neurodevelopmental morbidities and learning disorders that become apparent at school age, with anxiety and ADHD being the most commonly diagnosed (Linnet et al., 2006; Chyi et al., 2008; Moster et al., 2008; Petrini et al., 2009; Lindstrom et al., 2011; Loe et al., 2011; Baron et al., 2012; Cheong and Doyle, 2012; Potijk et al., 2012; Berry et al., 2018).

Attention deficit hyperactivity disorder is characterized by a deficit in behavioral inhibition, inattention, impulsivity and social difficulties, and in a Norwegian cohort of preterm/low birth weight children at 5 years old was more commonly diagnosed in males (Elgen et al., 2014). In the same cohort, the females were more likely to be diagnosed with anxiety (Elgen et al., 2014) highlighting that the behavioral outcomes of preterm birth occur in a sex-dependent manner. In a large Danish cohort children born at 34–36 weeks’ gestation (moderate-late preterm range) had an 80% increased risk of being diagnosed with ADHD compared to children born after 37 weeks’ gestation, a larger percentage of these were also male (Linnet et al., 2006). Furthermore, in a Swedish cohort, the amount of ADHD medication purchased for ex-premature school-aged males was more than three times as much than for females, and the amount purchased increased by degree of immaturity at birth (Lindstrom et al., 2011). In addition to anxiety and ADHD, incidences of autism and depression are also increased following preterm birth. Children in the United States that were born moderate-late preterm have been reported to have twice the incidence of autism at 10 years of age (Schendel and Bhasin, 2008). Parent-reported mental health rates in the United States are also higher for ex-preterm children than the general population for children and adolescents, with a prevalence of 22.9% compared to 15.5% in the general pediatric population (Singh et al., 2013). This study also revealed that ex-preterm children have 61% higher risk of having serious emotional/behavioral problems; specifically, a 33% higher chance of developing depression and a 58% higher chance of developing anxiety in childhood and adolescence (Singh et al., 2013).

School-related problems also arise in children following preterm birth, with those born preterm needing more special educational support, having an increased risk of repeating a grade and lower overall reading and mathematics scores compared to children born at full term (Chyi et al., 2008). These findings appear to be consistent internationally with numerous cohort studies observing that moderate-late ex-premature children have a 1.3- to 2.8-fold increased risk for requiring special education, and a 1.3- to 2.2-fold increased risk of repeating grades at ages 5–10 (Huddy et al., 2001; Morse et al., 2009; van Baar et al., 2009; Gurka et al., 2010). Furthermore, another study identified reading, writing, and spelling difficulties in 9- to 11-year-old ex-premature children compared to those born full term (Kirkegaard et al., 2006). Even at just 3–4 years of age impairments to visuospatial processing, spatial working memory, and sustained attention have been documented following preterm birth where major neurological deficits were not present at the time of birth (Vicari et al., 2004).

The direct impact of preterm birth on cognitive function is hard to quantify as it is confounded by many of the complex socio-economic, environmental and other factors that precipitated preterm birth in the first place. Additionally, given the plasticity of the developing brain, the timing of cognitive assessment needs to recognize the prognostic limitations of early assessment, especially for those born at extremes of gestational age. Studies comparing cognitive delays in toddlers at 2 years of age do not find any significant difference between preterm and term when corrected for prematurity (Cheatham et al., 2006; Darlow et al., 2009; Romeo et al., 2010; Woythaler et al., 2011). Alternatively, studies based in Swedish, American, and French cohorts found that 5- to 10-year old ex-premature children are twice as likely to score <85 on an IQ (intelligence quotient) test than term born children and that this is correlated with the gestational age at birth, with those being born more preterm at the highest risk of severe cognitive impairments (Schermann and Sedin, 2004; Marret et al., 2007; Talge et al., 2010). However, a much larger and comprehensive longitudinal American study in late-preterm ex-preterm 4- to 15-year olds found no significant differences in IQ based on 11 different cognitive tests at every age group (Gurka et al., 2010). These results suggest that intellectual disability may not be as prevalent following late-preterm birth as other negative outcomes such as behavioral disorders and poor school performance, suggesting that poor school performance may reflect behavioral disruptions that impact ability to pay attention and learn during class, rather than a result of reduced cognitive capacity.

## Exposure to the *ex utero* Environment and Associated Damage

Preterm birth abruptly removes the newborn from the supportive *in utero* environment experienced by a fetus of the same post-conceptional age. Organ maturation and function throughout the body changes tempo dramatically in response to this premature separation from the maternoplacental unit. Brain development requires neurotrophic and gliotrophic support during this time and so is vulnerable following preterm birth as it loses placental steroid support, the supply of precursors for fetal neurosteroid production, and other placentally supplied nutrients. In addition, premature loss of these steroids exposes the developing brain to increased stimulation and excitotoxic damage. Damage to oligodendrocytes of the developing nervous system can occur during this vulnerable developmental window prior to term gestation.

The oligodendrocyte development lineage is sensitive to premature exposure to the external environment, leading to injury by chemical and mechanical damage. This involves increased levels of reactive oxygen species following the rise in excitation after preterm birth and early exposure to the *ex utero* environment (Antony et al., 2004; Blasko et al., 2009). Demyelinated regions in relapsing remitting multiple sclerosis undergo remyelination but residual impaired motor coordination may remain (Dutta et al., 2011). Similarly, after preterm birth myelination continues and animal studies have shown less marked deficits at the equivalent of childhood, compared to the reduced myelination seen at term equivalent age. Despite this ‘catch up’ *ex utero* myelination, these children experience impaired learning ability and motor coordination, suggesting a similar causal pathway (Rees and Inder, 2005). Furthermore, reductions in myelination are apparent in a rat model of ADHD (Lindahl et al., 2008), and decreases in the white matter volumes of vulnerable regions such as the hippocampus and cerebellum are evident on magnetic resonance imaging (MRI) comparing term and preterm neonates (Counsell et al., 2003).

## Treatment Options for Preventing Poor Neurological Outcomes

In an effort to ameliorate or prevent preterm-associated brain damage a number of therapies have been adopted. However, despite the increasing body of evidence highlighting the increased neurodevelopmental vulnerability at all gestational ages below full term (39–40 weeks’ gestation) no targeted therapies are available in the perinatal period to those infants born late preterm (34–36 weeks). For the less mature infants, maternal magnesium sulfate has been shown to reduce cerebral palsy in extreme preterm neonates, but the number needed to treat remains high, highlighting the need for other therapeutic approaches. A Cochrane systematic review of five large trials comprising 6,145 babies found that the incidence of cerebral palsy in preterm neonates dropped from 5 to 3.4% following antenatal magnesium sulfate therapy (Doyle et al., 2009). A recent study where magnesium sulfate was given between 6 days and 12 h before unilateral hypoxic ischemia in neonatal rats identified that maximal neuronal protection was achieved by treatment only 24 h before the insult (Koning et al., 2017), which may be sufficient in some instances of preterm birth. Although promising, a limitation of this therapy is the need for antenatal rather than postnatal treatment, especially considering that more than 50% of preterm births are spontaneous and thus antenatal therapy cannot be initiated with appropriate timing. Human and animal studies have demonstrated a lack of neurological improvement following postnatal magnesium sulfate therapy in the context of chorioamnionitis induced preterm birth and asphyxia associated with preterm labor (Kamyar et al., 2016; Galinsky et al., 2017), thus although magnesium sulfate offers some therapeutic benefit, it is not, in itself sufficient to reduce preterm-related morbidity and rather may be suitable as an adjunct therapy.

The antioxidant melatonin has also been investigated for its neuroprotective benefits in animal models due to its roles in modulating neuroinflammation and reducing reactive oxygen species (Colella et al., 2016). In neonatal stroke and hemorrhage rat models pre-treatment with melatonin reduced the neuroinflammation and damage associated with stroke, whilst post-treatment reduced the amount of tissue death and improved cognitive and sensorimotor outcomes (Lekic et al., 2011; Villapol et al., 2011). However, despite entering clinical trials there are few conclusive results available, with a Cochrane systematic review finding no randomized trials published as yet (Wilkinson et al., 2016). Therefore, the long-term benefit of this treatment for neurobehavioral outcomes awaits the result of further randomized control trials.

Controlled therapeutic hypothermia in late preterm and term infants with hypoxic ischaemic encephalopathy has demonstrated well-established benefits, such as reduced mortality and decreased long-term neurodevelopmental disability, if implemented within 6 h of the insult occurring (Jacobs et al., 2013; Laptook, 2017). The physiological instability and vulnerability of the preterm infant means that therapeutic hypothermia is unlikely to be an appropriate intervention in this cohort. Even a small decrement in gestational age (to 34–35 week GA infants with HIE) at initiation of hypothermia was associated with an increase in over-cooling (Laptook, 2017) and other hypothermia-associated complications in 90% of the preterm group versus 81.3% in the term cohort (Rao et al., 2017). In this small retrospective cohort study, 66.7% of the preterm neonates that received hypothermia therapy had evidence of white matter injury, whilst just 25% of the term neonates with HIE showed signs of white matter injury following an asphyxial insult managed with therapeutic hypothermia. These results are difficult to interpret, however, due to the lack of a normothermic preterm-control group. Importantly, all deaths following the hypothermia therapy were in the preterm group, highlighting their increased vulnerability compared to the term neonates. Similarly, a small retrospective cohort analysis between 2007 and 2015 of preterm infants 33–35 weeks’ gestation who received whole body hypothermia revealed that 50% experienced mortality or moderate to severe neurodevelopmental impairment as a result of the therapy (Herrera et al., 2018). Currently there is an ongoing clinical trial implementing whole-body cooling in American preterm neonates born at 33–35 GA with moderate to severe neonatal encephalopathy, but as it is still in the recruiting stage results are not yet available (*ClinicalTrials.gov Identifier: NCT01793129*). The American Academy of Pediatrics committee advises that hypothermia should not be undertaken on preterm neonates due to the associated risks, unless it is performed in a research setting (Committee et al., 2014). These findings suggest that hypothermia may limit key developmental processes in the immediate postnatal period and this may limit its use in all but late preterm neonates, pending the outcome of the current clinical trial. Thus, the development of further adjunct therapy seems essential to improving neurodevelopmental outcome in the preterm infant.

## Placental Contribution to *in utero* Brain Development

**FIGURE 1:**
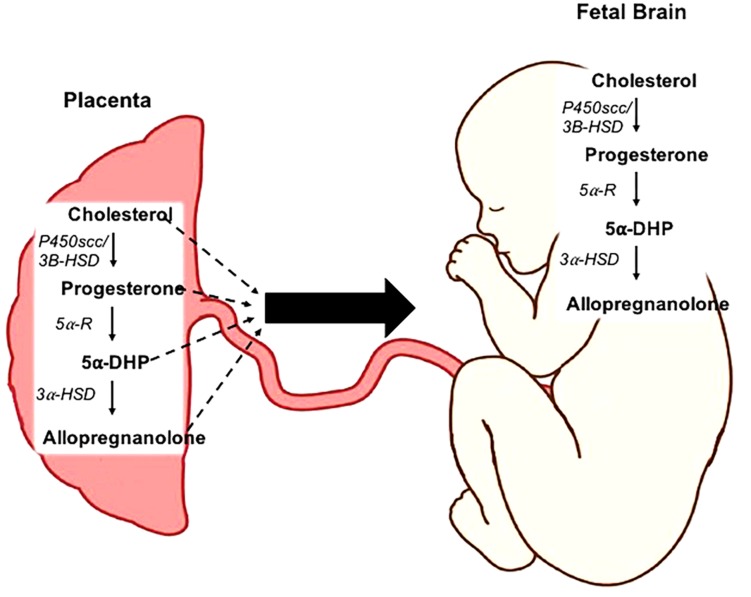
Neurosteroidogenesis in the placenta and fetal brain. Cholesterol is metabolized into progesterone by the enzymes cholesterol side-chain cleavage (P450scc) and 3β-hydroxysteroid dehydrogenase (3β-HSD). The rate-limiting enzymes 5α-reductase type 1 and 2 (5α-R) facilitate the conversion of progesterone into 5α-dihydroprogesterone (5α-DHP). Allopregnanolone is then synthesized from this precursor by 3α-hydroxysteroid dehydrogenase (3α-HSD). This process can occur both within the placenta, and *de novo* within the fetal brain.

The placenta plays an essential role in ensuring fetal neurodevelopment occurs correctly by secreting growth regulating factors including neurosteroid hormones throughout pregnancy (Figure 1). Neurosteroids are endogenous steroids that rapidly alter neuronal excitability through interaction with ligand-gated ion channels and other cell surface receptors. In late gestation, the fetus is maintained in a ‘sleep-like’ state, characterized by low levels of arousal-like activity. This ensures that excitation of the brain is limited, engendering a level of protection from excessive excitation and ultimately allowing sufficient energy for demanding developmental processes such as myelination to occur (Nicol et al., 1998; Nguyen et al., 2003). This fetal ‘sleep’ state is maintained by an elevated level of the neurosteroid allopregnanolone, and decreasing the synthesis of this neurosteroid has been shown to increase the excitation of the brain, potentially disrupting or delaying brain developmental processes (Yawno et al., 2007; Kelleher et al., 2011b). A reduction in the normal fetal neurosteroid environment is thus associated with adverse outcomes, such as the occurrence of potentially damaging seizures which can lead to destructive and permanent alterations in neurodevelopment (Yawno et al., 2011). Following preterm birth there is a premature reduction in the supply of neurosteroids, including progesterone and its neuroactive metabolite allopregnanolone, resulting in an already vulnerable premature neonate being exposed to the *ex utero* environment without neuroprotection.

### Importance of the Fetal Neurosteroid Allopregnanolone for Brain Development

Reductions in white matter is suggested to be a key component in the development of neurobehavioral disorders in children that are born preterm (Rees and Inder, 2005) and may stem from the birth-associated loss of allopregnanolone, as the pro-myelinating effects of this neurosteroid are evident *in vitro* on rat cerebellar slice cultures (Ghoumari et al., 2003). Allopregnanolone induced protection against cell death has been demonstrated in an *in vivo* mouse model of neurodegeneration (Liao et al., 2009) and in a sheep model of acute fetal hypoxia which is also important in maintaining levels of mature myelination oligodendrocytes (Yawno et al., 2007). Allopregnanolone is metabolized by the rate limiting enzymes 5α-reductases type 1 and 2 (5αR1 and 2) from progesterone (Figure 1) (Martini et al., 1996; Mellon and Griffin, 2002). In addition to the allopregnanolone supplied by the placenta to the fetus, the fetal brain is also capable of metabolizing allopregnanolone from placentally derived precursors including progesterone and 5α-dihyroprogesterone (5α-pregnane-3,20-dione), thus there is also a high level of allopregnanolone locally produced and maintained within the fetal brain (Stoffel-Wagner, 2001; Nguyen et al., 2004). However, we have previously shown in the developmentally relevant guinea pig (Morrison et al., 2018), a precocial species with similar hormonal profile to humans throughout pregnancy, that following the loss of the placenta at birth both progesterone and allopregnanolone levels decline rapidly within 24 h, highlighting the necessity of the placenta for the supply of steroidogenic precursors (Kelleher et al., 2013). Both of the rate-limiting enzymes 5αR1 and 2 are expressed in the placenta, and sheep and rat studies show that the 5αR2 isoform is most strongly expressed on neurons and glia within the developing fetal brain in late gestation (Martini et al., 1996; Nguyen et al., 2003).

Birth-associated loss of gestational allopregnanolone concentrations occurs earlier than normal in neonates that are born preterm leading to a damaging increase in excitation. Recent studies by our group have shown there is a dramatic drop in brain allopregnanolone concentrations following term and preterm birth compared to fetal levels (Kelleher et al., 2011a, 2013). Furthermore, preterm delivered animals also had significantly decreased myelination (evidenced by reduced MBP expression) in the CA1 region of the hippocampus and adjacent subcortical white matter 24 h after delivery compared to animals delivered at term (Kelleher et al., 2013). We have also shown that preterm male and female neonates at term equivalence age exhibit deficits in MBP immunostaining of the CA1 region, subcortical white matter and posterior lobe of the cerebellum (Kelleher et al., 2013; Palliser et al., 2015), and that juvenile offspring present with lasting deficits in myelination of these regions in male and female guinea pigs (Shaw et al., 2016, 2017). Likewise, reduced allopregnanolone supply as a result of intrauterine growth restriction and also impairs myelin development of the CA1 in male fetuses (Cumberland et al., 2017b). We have found that the late developing cerebellum is particularly vulnerable to the insults associated with preterm delivery. In addition to the CA1 region of the hippocampus, reductions in myelination of the posterior lobe of the cerebellum were evident in preterm guinea pig neonates at PND1 (Shaw et al., 2015). Furthermore, at term equivalence age we have demonstrated that not only is the expression of the level of mature oligodendrocytes reduced, but also that reductions are present throughout the oligodendrocyte lineage thereby lessening the potential of catch-up growth to occur (Palliser et al., 2015). By juvenile age we further observed that there were sex dependent alterations in myelination of the posterior lobe of the cerebellum as well as in components of the GABAergic pathway (Shaw et al., 2017). Functional imaging studies suggest that the posterior lobe of the cerebellum is particularly involved in cognition and emotion (Stoodley, 2012), as it is interconnected with the prefrontal cortex, association cortices, and the limbic system, which allows for its involvement in higher order executive functioning (Stoodley and Schmahmann, 2010). Therefore, the altered development of this area may be having a role in some of the neurobehavioral disorders that are more common following premature birth, such as ADHD and autism.

Our studies indicate that juvenile males show a hyperactive phenotype following preterm birth (Shaw et al., 2016). Additionally, they exhibit behavior similar to that observed in mouse models of ADHD where, as with our study, within open field test conditions the spontaneous distance traveled, and time spent mobile is markedly higher for the affected mice compared to the controls (Kim et al., 2014). This hyperactive behavior has parallels with clinical studies where ex-preterm male children show an increased incidence of hyperactivity disorders (Linnet et al., 2006; Lindstrom et al., 2011). Taken together, these data emphasize the importance of allopregnanolone for myelination and optimal development of the GABAergic system to occur in fetal and neonatal life. We therefore speculate that the changes in neurodevelopmental and behavioral function we see following preterm birth may be accounted for by the loss of allopregnanolone supply.

### Pharmacological Reduction of the *in utero* Neurosteroid Environment

**FIGURE 2:**
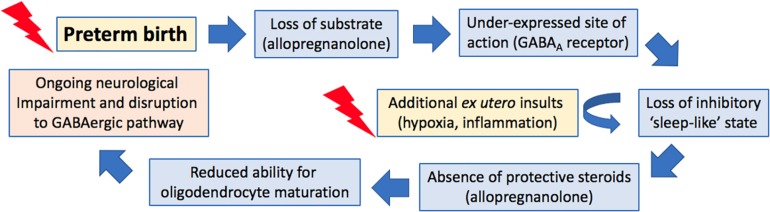
Proposed cascade of events following preterm birth that lead to ongoing neurological impairments.

The deficits in myelination seen following preterm birth can be mimicked by the administration of a 5α-reductase inhibitor, finasteride, directly to the fetal circulation preventing the metabolism of progesterone to allopregnanolone within the fetal brain. This intervention results in an increase of damaging excitation in the brain of fetal sheep due to reduced suppression by allopregnanolone (Nicol et al., 2001). As a result of this excitation, cell death is increased in areas such as the hippocampus, cerebellum, and white matter tracts. In another study in fetal sheep, allopregnanolone synthesis was reduced through inhibition of progesterone production by trilostane (a 3β-hydroxysteroid dehydrogenase inhibitor). This resulted in reduced fetal sleep-like behavior but increased arousal-like activity (Crossley et al., 1997), resulting in increased brain excitability and damaging seizures (Mirmiran, 1995; Nicol et al., 1997). Furthermore, when progesterone was replaced by exogenous supplementation, the occurrence of sleep-like behavior returned to normal fetal patterns (Crossley et al., 1997). Exposure to finasteride has also been shown to increase apoptotic cells in the CA1 and CA3 regions of the hippocampus, and the cerebellar molecular and granular layers in fetal sheep, as well as increasing the number of dead Purkinje cells in the cerebellum (Yawno et al., 2009). Importantly, co-infusion of finasteride and the allopregnanolone analog alfaxolone completely ameliorated the deleterious effects of finasteride treatment. Similarly allopregnanolone itself has also been shown to protect the fetal brain when insults occur, in a sheep model the introduction of brief asphyxia in the presence of finasteride induced cell death in the hippocampus, however, when allopregnanolone was present in normal concentrations this asphyxia- induced damage did not occur (Nicol et al., 2001). *In utero* administration of finasteride to guinea pigs has also highlighted the key role of allopregnanolone in myelination, as a reduction in myelination in the subcortical white matter was present following inhibition of allopregnanolone synthesis (Kelleher et al., 2011b). Interestingly, administration of the allopregnanolone precursor, progesterone, to *in vitro* rat cerebellum slices increased both the proliferation of myelinating oligodendrocytes and the rate of myelination (Ghoumari et al., 2003). Follow up studies then revealed that this effect was achieved by neurosteroids acting on the GABA_A_ receptors (Ghoumari et al., 2005). Together these studies emphasize the important role of allopregnanolone in not just the development of the brain, but also for protection from hypoxia (Figure 2).

A reduction in allopregnanolone concentrations during pregnancy can also have long-lasting effects on the offspring. In guinea pigs, late gestation maternally administered finasteride resulted in an anxiety-like phenotype in female offspring, along with reductions in components of the GABAergic pathway within the amygdala (Cumberland et al., 2017a). Furthermore, there was also decreased expression of neurosteroid-sensitive GABA_A_ receptors and increased astrocyte activation within the cerebellum of these animals (Cumberland et al., 2017c). In a similar study, finasteride treatment to pregnant rats during late gestation resulted in increased serum corticosterone concentrations in their juvenile offspring, decreased hippocampal allopregnanolone levels and impaired performance in memory tasks (Paris et al., 2011). Studies inhibiting the production of allopregnanolone in adult rats highlight the importance of allopregnanolone for the prevention of neurodevelopmental disorders throughout life as reductions in the concentration of allopregnanolone within the hippocampus (Frye and Walf, 2002) or the amygdala (Walf et al., 2006) increased anxiety-like behaviors in these animals. Furthermore, multiple neurological conditions are characterized by a reduced level of circulating allopregnanolone in adults, including post-traumatic stress disorder (Rasmusson et al., 2006), major depressive disorder (Strohle et al., 1999), and premenstrual dysphoric disorder (Monteleone et al., 2000; Lombardi et al., 2004).

### Combined Effect of Reduced Neurosteroid Exposure and Increased Cortisol

An underlying factor involved in the development of hyperactivity and anxiety following preterm birth may be increased cortisol. In our studies we have observed increased circulating cortisol levels in preterm offspring at birth (Shaw et al., 2015), PND1 (Shaw et al., 2015), and juvenility (Shaw et al., 2016). In humans, one study has found that as birth weight and gestational age decreases, there is an increase in circulating cortisol (Kajantie et al., 2002), and early life stress has also been shown to negatively impact hippocampal development with long-term effects into adolescence (Hodel et al., 2015). Interestingly at juvenility, we found that male preterm offspring had increased baseline concentrations of circulating cortisol that were unaffected by exposure to foreign situations (in the form of behavioral testing). Meanwhile, juvenile females born preterm experienced a substantial rise in cortisol in response to foreign situations compared to term-born females, suggesting that they have an anxious phenotype and increased fear response (Shaw et al., 2016). These data highlight the sexually dimorphic effects that preterm birth has on programming of the hypothalamic pituitary axis, with a blunting of the stress response following preterm birth in males, but an increased response in females. Previous studies in guinea pigs suggest that prenatally increased cortisol may program adverse behavior in childhood, for example maternal stress exposure in pregnancy was shown to result in increased anxious behaviors in juvenile female offspring (Bennett et al., 2015). This is consistent with studies showing that prenatal stresses ‘programs’ the HPA axis (Kapoor and Matthews, 2005, 2008; Kapoor et al., 2006). This results in a greater postnatal sensitivity of the HPA axis to stressful stimuli, in turn contributing to behavioral disorders. The programming mechanism has been shown to be mediated by changes at the level of the hypothalamus (Kapoor and Matthews, 2005, 2008; Kapoor et al., 2006). Therefore, even in the absence of a parallel change in postnatal cortisol concentrations, early exposure to increased cortisol concentration can program an altered behavioral response to stress-inducing situations.

These behavior-altering effects of cortisol may also involve interactions between cortisol and allopregnanolone. Glucocorticoids, such as cortisol, are known to adversely affect allopregnanolone production. Studies in guinea pigs have previously demonstrated that repeated administration of betamethasone (a synthetic glucocorticoid) to pregnant dams reduced the allopregnanolone synthesizing capacity of both the placenta and the fetal brain as demonstrated by a reduction in the expression of the rate-limiting enzyme 5α-reductase type 2 in both tissues (McKendry et al., 2009). Interestingly expression of this enzyme is also decreased in the brain of preterm guinea pig neonates (Kelleher et al., 2013), possibly as a result of exogenous glucocorticoid exposure, part of the gold standard treatment to reduce short-term morbidity and mortality following preterm birth. Our studies have also shown that both late gestation maternal stress and pharmacological inhibition of allopregnanolone synthesis by finasteride result in a reduction of allopregnanolone concentrations in the fetus, with development of an anxious phenotype in female juvenile offspring (Bennett et al., 2015; Cumberland et al., 2017a). In light of these data and the findings of the studies presented here we suggest that in addition to the lack of protection of allopregnanolone against excitotoxic damage, and the raised levels of cortisol present following early exposure to the *ex utero* environment, that cortisol hinders the synthesis and action of any offspring derived allopregnanolone in the preterm neonate and that this has lasting implications on neurodevelopment and behavior (Figure 2).

## Neurosteroids and the Extra Synaptic Gaba_A_ Receptor

Inhibitory allopregnanolone exert effects throughout the brain to suppress excessive excitation. This suppression is achieved by increasing GABAergic inhibition (Herd et al., 2007). Allopregnanolone is an allosteric agonist of the GABA_A_ receptors and specifically enhances GABA_A_ receptor mediated inhibition, which results in anxiolytic, anti-convulsant, anesthetic, analgesic, and sedative effects (Harrison and Simmonds, 1984; Harrison et al., 1987; Lambert et al., 1987; Majewska, 1992; Paul and Purdy, 1992; Olsen and Sapp, 1995; Belelli and Lambert, 2005). These effects are achieved by activation of the extra synaptic receptors, which are known to be particularly sensitive to allopregnanolone. GABA_A_ receptors form a gated chloride ionophore channel and specific binding sites for benzodiazepines, barbiturates, and anesthetics, however, neurosteroids are thought to bind to a separate allosteric steroid-binding site (Delaney and Sah, 1999; Macdonald and Botzolakis, 2009). GABA_A_ receptors exhibit inhibitory effects in response to neurosteroid stimulation in adult animals and from mid gestation onward in the fetus, however, they are also capable of exhibiting excitatory actions in early gestation and these excitatory actions are known to stimulate glial cells and neuronal outgrowth (Owens and Kriegstein, 2002; Represa and Ben-Ari, 2005). Whether the effect is inhibitory or excitatory is determined by the chloride gradient of the receptor-ionophore, determined by the intracellular chloride concentration. This in turn is primarily regulated by the K^+^/Cl^–^ co-transporter-2 (KCC2) (Rivera et al., 1999, 2005). The expression and activity of this integral co-transporter is regulated by the phosphorylation of its Ser940 residue, with dephosphorylation resulting in downregulation of the co-transporter, increasing the intracellular chloride concentration, and switching to excitatory GABA actions (Lee et al., 2007; Lee et al., 2011).

GABA_A_ receptors are involved in a broad range of functions including controlling the excitability of the brain, modulation of anxiety, as well as cognition, memory, and learning (Sieghart et al., 1999). In addition to neurons, extra synaptic neurosteroid sensitive receptors are highly expressed on glial cells including oligodendrocytes (Arellano et al., 2016) throughout the fetal brain from mid-gestation onward (Williamson et al., 1998; Crossley et al., 2000; Hirst et al., 2008). The expression of GABA_A_ receptors in the fetal brain increases as gestation advances, reaching their highest levels of expression by full term gestation in most areas, such as the cerebral cortex and hypothalamus (Crossley et al., 2000, 2003; Nguyen et al., 2003). GABA_A_ receptors exist in a pentameric formation of 5 subunits with a central selective chloride anion channel. The five subunits come from a pool of 19 different subunits, α1-6, β1-3, γ1-3, δ, ε, π, θ, and ρ1-3 and subunit composition varies greatly depending on the function of the receptor (Barnard et al., 1998; Belelli et al., 2009). Synaptic receptors, which are responsible for fast transmission, usually feature the α1-3, β1-3, and γ2 subunits (Essrich et al., 1998), whilst the extra synaptic receptors that contribute to tonic inhibition (Belelli et al., 2009) possess the α4-6 and δ subunits (Burgard et al., 1996). Rather than produce an increase in amplitude of miniature inhibitory postsynaptic currents (mIPSCs), neurosteroids have been shown to increase the duration of the amplification by altering the kinetics of the GABA_A_-gated ion channels (Lambert et al., 2003). This increase in duration is neuron specific, with different brain regions requiring different concentrations of neurosteroids to induce the same effect. Specifically, the CA1 neurons of the hippocampus, cerebellar granule cells, and Purkinje cells appear to be more sensitive to neurosteroids, only requiring low nanomolar concentrations to increase duration of amplification (Harney et al., 2003; Cooper et al., 2004), and this is primarily due to subunit composition.

Receptor subunit composition plays an important role in determining receptor affinity for various ligands. Benzodiazepines for example are known to be attracted to receptors containing a γ subunit, whilst those featuring α6 are unresponsive to benzodiazepines (Delaney and Sah, 1999). Whilst there is a specific binding site for 3α-hydroxy-neurosteroids such as allopregnanolone, the composition of subunits affects the sensitivity of the receptor to stimulation (Belelli et al., 2002; Hosie et al., 2007). Regional specificity also exists for these receptors, for example in a mouse knockout of the δ subunit tonic conductance was significantly reduced in the cerebellum, however, in the CA1 region of the hippocampus there was no effect on conductance (Stell et al., 2003). This regional specificity is due to differences in expression of various subunits throughout the brain and whilst the α6 and δ subunits, which are co-expressed in many receptors, are highly expressed in the cerebellum, tonic conductance in the hippocampus is controlled primarily by receptors containing the α4 and α5 subunits, in addition to those containing the δ subunit.

The role of specific neurosteroid-sensitive subunits in behavior has been revealed in knockout mouse models. For example, global deletion of the δ subunit significantly reduces the anxiolytic and anti-convulsant effects induced by the allopregnanolone analog ganaxolone, confirming that neurosteroids bind to the δ subunit containing GABA_A_ receptors to exert their inhibitory functions (Mihalek et al., 1999). Increased anxiety-like behavior was also present in a α4 subunit knockout mice as demonstrated by an increased preference for dark enclosed spaces in a T-maze (Loria et al., 2013). Seizure susceptibility has also been shown as increased following this knockout (Chandra et al., 2008). Similarly, it has been demonstrated that pro-epileptic behavior is increased in mice lacking the δ subunit (Mihalek et al., 1999; Spigelman et al., 2002, 2003). Taken together these data indicate the importance of configurations of the GABA_A_ receptors and the necessity of the expression of key subunits for neurosteroid binding and for their effects on behavior.

Of particular importance to preterm-associated neurodevelopmental impairment is the ability of allopregnanolone to promote GABA_A_ receptor-mediated maturation of oligodendrocytes. Administration of progesterone to rat cerebellar slice cultures increased the expression of the mature myelinating oligodendrocyte marker, myelin basic protein (MBP) (Ghoumari et al., 2003). The enhancement of myelination was achieved by allopregnanolone, the neuroactive metabolite of progesterone, acting via the GABA_A_ receptors located on oligodendrocytes as a selective GABA_A_ receptor antagonist inhibited this promyelinating effect.

## GABA_A_ Receptors and Preterm Birth

**FIGURE 3:**
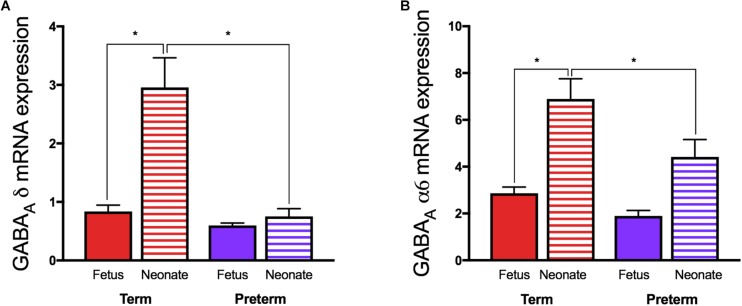
Relative mRNA expression of the GABA_A_ receptor **(A)** δ and **(B)** α6 subunits in guinea pig cerebellum. Fetal tissue was obtained at GA69 (term) and GA62 (preterm) ages, and neonatal tissue from 24 h after term or preterm birth. (*p < 0.05, *n* = 11–16). Adapted and reprinted with permission from Shaw et al. (2015).

In juvenile guinea pigs born preterm, altered GABAergic pathway development is evident at juvenility in the cerebellum. Intriguingly, despite reduced expression of both subunits in the preterm neonatal cerebellums (Figure 3) (Shaw et al., 2015) mRNA expression of allopregnanolone sensitive GABA_A_ receptor subunits α6 and δ are not altered in these preterm-born animals at juvenility (Shaw et al., 2017). These observations suggest that sometime between birth and juvenile age in the guinea pig (PND28) there is either a ‘catch-up’ in these key GABA_A_ receptor subunits expression, or conversely, that levels in the brain of term born animals have dropped to lower levels. Subunits of the GABA_A_ receptor are reported to go through age-related changes in expression, with early development often a period of high expression, followed by down regulation in adulthood (Yu et al., 2006). This age-related change in expression follows the maturation profile of the brain and therefore if the neurosteroid-sensitive receptors in the preterm brain do not undergo any ‘catch-up’ between birth and juvenility this may contribute to preterm-associated changes in neurodevelopment. An additional vulnerability that has been reported for preterm neonates is an observed lack of a birth-related adaptive increase in the cerebellar expression of the α6 and δ GABA_A_ receptor subunits after birth (Figure 2) (Shaw et al., 2015). This potentially reduces the effect of allopregnanolone postnatally, exposing the immature brain to damaging excitotoxicity. Knockout studies of the δ subunit, which is known to commonly group with the α6 subunit, have shown a link between a lack of these subunits with the manifestation of multiple neurodevelopmental phenotypes such as anxiety-like behavior and pro-epileptic behavior (Mihalek et al., 1999; Spigelman et al., 2002, 2003). Interestingly, receptor changes are present in human brain tissue in disorders that primarily affect myelination, such as multiple sclerosis (Luchetti et al., 2011), and whilst their precise role in disease progression is unknown, the neurosteroid sensitive GABA_A_ receptors present a common link between initial myelination, ‘catch up’ and remyelination processes, and behavioral state.

Conversely, the hippocampal GABA_A_ neurosteroid sensitive receptor subunits appear to be largely unaffected by preterm delivery with the exception of a decrease in the expression of the α5 subunit mRNA at juvenility (Shaw et al., 2016). This particular subunit is known to mediate tonic inhibition in the CA1 of the hippocampus, is required for associative learning and furthermore is known to be reduced in response to increased levels of cortisol (Crestani et al., 2002; Verkuyl et al., 2004; Glykys and Mody, 2006). Thus, a reduction in α5 subunit expression in childhood may reduce tonic inhibition, thereby increasing excitation in the hippocampus, which in turn may contribute to the risk of hyperactivity-disorders in male children born preterm.

## Potential of Neurosteroids as a Protective Therapy

Steroid hormones, including progesterone, allopregnanolone and potentially other neuroactive metabolites, can exert neuroprotective effects following damage to neurons and glia by preventing excitation, apoptosis, and inflammation, as well as by regenerative mechanisms (Schumacher et al., 2004). Studies in adult rats have demonstrated the therapeutic effect of progesterone injections on TBI where progesterone administration reduced neuronal loss (Roof et al., 1994, 1996; He et al., 2003). Similarly, allopregnanolone administration was shown to reduce memory deficits and loss of neurons in the frontal cortex of these rats following bilateral injury by stimulating trophic effects (He et al., 2003). Importantly, in rat astrocytes and oligodendroglial progenitor primary cell cultures, progesterone exposure upregulated expression of the promyelinating factor insulin-like growth factor 1 (Chesik and De Keyser, 2010) and, in organotypic slice cultures of rat cerebellum, myelination was stimulated by progesterone following its metabolism into allopregnanolone and its’ trophic actions mediated by actions on GABA_A_ receptors (Ghoumari et al., 2003). Both progesterone and allopregnanolone have been shown to be effective at reducing the pro-apoptotic activity of caspase-3, reducing astrogliosis as evidenced by GFAP staining, and improving performance in both the spatial learning task and memory function in adult male rats (Djebaili et al., 2005). Furthermore, rat studies have identified reductions in inflammatory cytokines TNF-α and IL-1β following TBI and subsequent progesterone or allopregnanolone administration (He et al., 2004). Following the potential benefits of progesterone therapy observed in animal studies, a randomized phase III clinical trial of progesterone (ProTECT) for treatment of acute TBI in adults was performed. This showed that progesterone treatment resulted in a lower 30-day mortality risk, and that patients were more likely to have a moderate to good outcome than those receiving placebo (Wright et al., 2007). Likewise, a large clinical trial in China is showing similar therapeutic benefits following progesterone therapy (Xiao et al., 2008).

The role of progesterone as a precursor of allopregnanolone, and the number of positive studies relating to the use of progesterone, led to us examining the use of progesterone replacement therapy in preterm guinea pig neonates. In contrast to the earlier finding of effects on TBI in rats, we observed detrimental effects on postnatal neurodevelopment particularly in the male offspring. From this preliminary study, it appears that progesterone is metabolized differently by the male neonates and instead of producing allopregnanolone, much of the steroid is converted to cortisol. These males, with high plasma and salivary cortisol concentrations, also had reductions in myelination of the cerebellum and subcortical white matter, highlighting the vulnerability of these male neonates to increased cortisol as a result of increased postnatal progesterone (Palliser et al., 2015).

Previous studies have also investigated the potential use of allopregnanolone to restore neurosteroid deficits. Preliminary findings, however, suggested that allopregnanolone had limited effectiveness due to the very short half-life of allopregnanolone, or other possible metabolic conversion making therapeutic concentration difficult to achieve. To avoid both of these issues with allopregnanolone therapy, as well as potential conversion of allopregnanolone to its less active isomers, we explored a possible postnatal therapy with ganaxolone.

### Ganaxolone

Ganaxolone is a 3β-methylated synthetic analog of allopregnanolone initially developed by Edward Monaghan at CoSensys in 1998, however, in 2004 Marinus Pharmaceuticals Inc., acquired the development and commercialization rights (Nohria and Giller, 2007). Marinus Pharmaceuticals then carried out a number of clinical trials using ganaxolone, some of which are still ongoing. Ganaxolone features a methyl group that prevents metabolism into other active steroids (Carter et al., 1997), and a half-life of 12–20 h in humans (Monaghan et al., 1997). Ganaxolone acts in a very similar manner to allopregnanolone and binds to the neurosteroid-binding site of GABA_A_ receptors, producing similar anxiolytic and anti-seizure effects. The addition of the methyl group markedly improves oral pharmacokinetics and in addition ganaxolone is not readily metabolized to other steroids that may bind elsewhere and produce unwanted effects (Carter et al., 1997). Allopregnanolone can for example be metabolized into the 3β-isomer that is either inactive, or at higher doses, may block the steroid site on the GABA_A_ receptor.

Animal pharmacokinetic studies demonstrate that ganaxolone has a large volume of distribution as administration of radioactively labeled ganaxolone has shown wide distribution, and due to its’ lipophilic nature, it becomes concentrated in the brain with a brain-to-plasma concentration of between 5 and 10 (Nohria and Giller, 2007; Reddy and Rogawski, 2012). In addition to pharmacokinetic studies there have been a number of animal studies relating to the use of ganaxolone and behavioral disorders. In an adult mouse model of Angelman syndrome (which is characterized by severe developmental delay, motor impairments, and epilepsy) treatment with ganaxolone over a period of 4 weeks was shown to ameliorate behavioral abnormalities (Ciarlone et al., 2017). Other mouse models of neurodevelopmental disorders have highlighted the therapeutic benefits of ganaxolone, including an adult mouse model of autism where ganaxolone reversed the autistic phenotype (Kazdoba et al., 2016), and an adult post-traumatic stress mouse model where again ganaxolone therapy improved behavioral changes such as aggression and anxiety (Pinna and Rasmusson, 2014). Despite numerous animal models of behavioral disorders demonstrating the therapeutic potential of ganaxolone in ameliorating disease states, there is limited information regarding the effects on neurodevelopment or myelination in these models. There has been one model where administration of ganaxolone to Niemann-Pick Type C diseased adult mice identified protection against Purkinje cell death, which is similar to the previously reported protective mechanisms of allopregnanolone (Mellon et al., 2008). Furthermore, there has only been one neonatal animal study using ganaxolone therapy, in a rat model of infantile spasms where the onset, number, and duration of spasms were reduced by ganaxolone therapy (Yum et al., 2014). An additional study examining the neuroprotective effects of ganaxolone following neonatal seizures in sheep is ongoing but shows promise (Yawno et al., 2017).

A number of phase 2 clinical trials have examined the use of ganaxolone for epilepsy and infantile spasms, as well as for posttraumatic stress disorder, migraine, and the developmental problems associated with fragile X syndrome (Nohria and Giller, 2007; Reddy and Rogawski, 2012). Daily drug doses of up to 1,875 mg in adults and 54 mg/kg in children have been trialed, and it has been shown that a single oral dose of 1,600 mg can result in peak plasma concentrations of up to 460 ng/mL. Recently a randomized phase 2 trial for ganaxolone as an add-on therapy for severe seizure disorders took place in 147 adults (Sperling et al., 2017). The subjects received 1,500 mg/day spread over three doses for 8 weeks. The treatment resulted in an 18% decrease in mean weekly seizure frequency, compared to a 2% increase in the placebo group. The treatment was reported as safe and well tolerated with similar rates of discontinuation due to adverse effects in the placebo and ganaxolone groups (ganaxolone 7.1% versus 6.1% for placebo). The most common side effects were classified as mild to moderate and included dizziness (16.3% versus 8.2% in placebo), fatigue (16.3% versus 8.2%), and somnolence (13.3% versus 2.0%).

**FIGURE 4:**
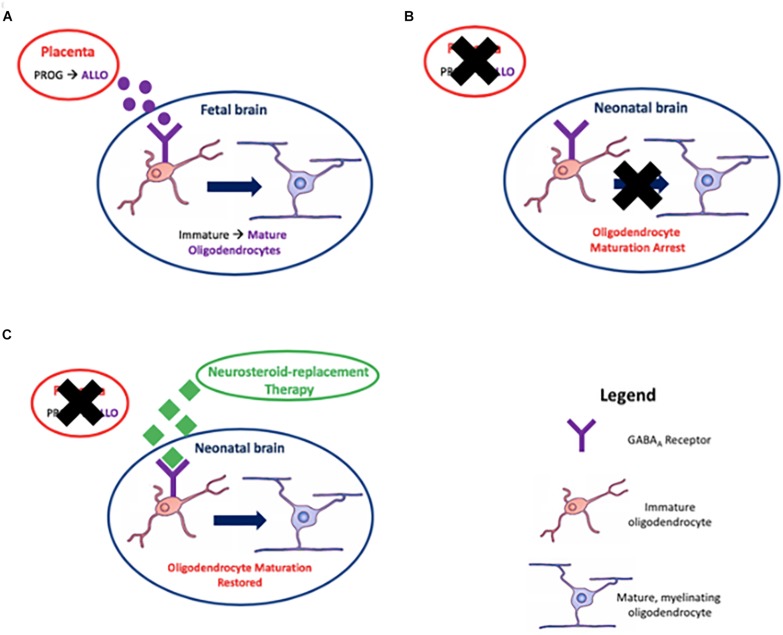
Myelination, the process of surrounding nerve axons with a myelin sheath, is achieved by oligodendrocytes. Placentally derived allopregnanolone (ALLO), the neuroactive metabolite of progesterone (PROG), promotes maturation of oligodendrocytes *in utero* via action on GABA_A_ receptors in **(A)**; premature loss of the placenta due to preterm birth results in an arrest in this process in **(B)**; and reinstating GABA_A_ receptor signaling by neurosteroid-replacement therapy may restore oligodendrocyte maturation leading to correct myelination in neonates born preterm, thus improving neurological function in **(C)**.

In the context of preterm birth, we have recently reported that ganaxolone neurosteroid-replacement therapy given to preterm guinea pigs between birth and term ‘due date’ improved myelination of the CA1 region of the hippocampus and overlying subcortical white matter, in addition to reduction in hyperactive behavior (Shaw et al., 2018). This was the first study to show that neurosteroid-replacement therapy can replicate the *in utero* neurosteroid environment and that this restores neurodevelopment to a normal, term-born, trajectory (Figure 4). By combining our recent studies on pregnancy compromises in the developmentally relevant guinea pig (Morrison et al., 2018) and the impact of disturbances in allopregnanolone levels on the developing fetus, the preterm neonate, and the long-term effects on the juvenile, we now suggest that re-establishment of neurosteroid action in the period between birth and term equivalence is a prospective therapy for future clinical use. Whilst more studies required, particularly on optimal dosing and longer-term outcomes, we suggest this study provides the impetus and a path for future preclinical trials using neurosteroid-replacement therapy following preterm birth. Furthermore, this therapy may be useful following other pregnancy compromises discussed previously where a major contributing factor to deficits in neurodevelopment is a lack of allopregnanolone exposure.

## Conclusion

Until recently, the risk of neurodevelopmental impairment in children born moderate-late preterm who required little to no clinical intervention, was thought to be minimal, however, data from large international cohorts clearly demonstrate that this is not the case. Albeit that the effect size is not as great as for those born at extremes of gestational age, the significantly larger number of children born at moderate-late preterm gestations means that this is an increasingly large public health issue, with important implications for the provision of educational and other resources throughout childhood. Currently, there are no targeted therapies available to prevent the development of these neurodevelopmental problems, and as such therapy is limited to symptom management for the most affected children.

Through use of studies in our model of preterm birth in the guinea pig we have begun to address these gaps in the knowledge of neurodevelopment following preterm birth. We suggest key pathways involved, targets for intervention, and a therapy for prevention of preterm-associated neurodevelopmental disorders. These studies are in their preliminary stages and whilst we have identified a target for improving outcomes, there are many aspects to this therapy that we are yet to investigate. Our pilot studies are primarily focused on identifying an optimal dose that promotes oligodendrocyte maturation but minimizes adverse side effects. Once we identify an ideal dose, we can then determine whether there are interactions with other therapies that the preterm neonate may be exposed to, such as synthetic glucocorticoids, and potentially in the future, for asphyxiated preterm infants, therapeutic hypothermia as a co-therapy.

## Author Contributions

JS: primary author. MB, RD, and GC: revisions and edits. JH and HP: co-senior authors, revisions and edits, and concept design.

## Conflict of Interest Statement

The authors declare that the research was conducted in the absence of any commercial or financial relationships that could be construed as a potential conflict of interest.

## References

[B1] Ananth C. V. Vintzileos A. M. (2006). Epidemiology of preterm birth and its clinical subtypes. *J. Matern. Fetal Neonatal Med.* 19 773–782. 17190687 10.1080/14767050600965882

[B2] Antony J. Van Marle G. Opii W. (2004). Human endogenous retrovirus glycoprotein-mediated induction of redox reactants causes oligodendrocyte death and demyelination. *Nat. Neurosci.* 7 1088–1095. 10.1038/nn1319 15452578

[B3] Arellano R. O. Sanchez-Gomez M. V. Alberdi E. Canedo-Antelo M. Chara J. C. Palomino A. (2016). Axon-to-Glia interaction regulates GABAA receptor expression in oligodendrocytes. *Mol. Pharmacol.* 89 63–74. 10.1124/mol.115.100594 26538574

[B4] Arnold S. E. Trojanowski J. Q. (1996). Human fetal hippocampal development: I. Cytoarchitecture, myeloarchitecture, and neuronal morphologic features. *J. Comp. Neurol.* 367 274–292. 8708010 10.1002/(SICI)1096-9861(19960401)367:2<274::AID-CNE9>3.0.CO;2-2

[B5] Back S. A. Han B. H. Luo N. L. Chricton C. A. Xanthoudakis S. Tam J. (2002). Selective vulnerability of late oligodendrocyte progenitors to hypoxia–ischemia. *J. Neurosci.* 22 455–463. 10.1523/jneurosci.22-02-00455.2002 11784790 PMC6758669

[B6] Barnard E. Skolnick P. Olsen R. Mohler H. Sieghart W. Biggio G. (1998). International Union of Pharmacology. XV. Subtypes of γ-aminobutyric acidA receptors: classification on the basis of subunit structure and receptor function. *Pharmacol. Rev.* 50 291–314. 9647870

[B7] Baron I. S. Litman F. R. Ahronovich M. D. Baker R. (2012). Late preterm birth: a review of medical and neuropsychological childhood outcomes. *Neuropsychol. Rev.* 22 438–450. 10.1007/s11065-012-9210-5 22869055

[B8] Belelli D. Casula A. Ling A. Lambert J. J. (2002). The influence of subunit composition on the interaction of neurosteroids with GABA_A_ receptors. *Neuropharmacology* 43 651–661. 10.1016/s0028-3908(02)00172-7 12367610

[B9] Belelli D. Harrison N. L. Maguire J. Macdonald R. L. Walker M. C. Cope D. W. (2009). Extrasynaptic GABAA receptors: form, pharmacology, and function. *J. Neurosci.* 29 12757–12763. 10.1523/JNEUROSCI.3340-09.2009 19828786 PMC2784229

[B10] Belelli D. Lambert J. J. (2005). Neurosteroids: endogenous regulators of the GABAA receptor. *Nat. Rev. Neurosci.* 6 565–575. 10.1038/nrn1703 15959466

[B11] Bennett G. A. Palliser H. K. Shaw J. C. Walker D. Hirst J. J. (2015). Prenatal stress alters hippocampal neuroglia and increases anxiety in childhood. *Dev. Neurosci.* 37 533–545. 10.1159/000437302 26279160

[B12] Berry M. J. Foster T. Rowe K. Robertson O. Robson B. Pierse N. (2018). Gestational age, health, and educational outcomes in adolescents. *Pediatrics* 74 200–201. 10.1097/01.ogx.0000554436.92583.c7 30381471

[B13] Blasko I. Humpel C. Grubeck-Loebenstein B. (2009). *Astrocytes and Oligodendrocytes During Normal Brain Ageing.* Oxford: Academic Press.

[B14] Burgard E. C. Tietz E. I. Neelands T. R. Macdonald R. L. (1996). Properties of recombinant gamma-aminobutyric acid A receptor isoforms containing the alpha 5 subunit subtype. *Mol. Pharmacol.* 50 119–127. 8700104

[B15] Caldinelli C. Froudist-Walsh S. Karolis V. Tseng C. E. Allin M. P. Walshe M. (2017). White matter alterations to cingulum and fornix following very preterm birth and their relationship with cognitive functions. *Neuroimage* 150 373–382. 10.1016/j.neuroimage.2017.02.026 28216430 PMC5405171

[B16] Carter R. Wood P. J. Wieland S. (1997). Characterization of the anticonvulsant properties of Ganaxolone (CCD 1042; 3α-Hydroxy-3β-methyl-5α-pregnan-20-one), a selective, high-affinity, steroid modulator of the γ-aminobutyric acida receptor. *J. Pharmacol. Exp. Ther.* 280 1284–1295. 9067315

[B17] Chandra D. Werner D. F. Liang J. Suryanarayanan A. Harrison N. L. Spigelman I. (2008). Normal acute behavioral responses to moderate/high dose ethanol in GABAA receptor alpha 4 subunit knockout mice. *Alcohol Clin. Exp. Res.* 32 10–18. 10.1111/j.1530-0277.2007.00563.x 18076749 PMC2896280

[B18] Cheatham C. Bauer P. Georgieff M. (2006). Predicting individual differences in recall by infants born preterm and full term. *Infancy* 10 17–42. 10.1207/s15327078in1001_2 33412675

[B19] Cheong J. L. Doyle L. W. (2012). Increasing rates of prematurity and epidemiology of late preterm birth. *J. Paediatr. Child Health* 48 784–788. 10.1111/j.1440-1754.2012.02536.x 22970672

[B20] Chesik D. De Keyser J. (2010). Progesterone and dexamethasone differentially regulate the IGF-system in glial cells. *Neurosci. Lett.* 468 178–182. 10.1016/j.neulet.2009.10.051 19853640

[B21] Chyi L. J. Lee H. C. Hintz S. R. Gould J. B. Sutcliffe T. L. (2008). School outcomes of late preterm infants: special needs and challenges for infants born at 32 to 36 weeks gestation. *J. Pediatr.* 153 25–31. 10.1016/j.jpeds.2008.01.027 18571530

[B22] Ciarlone S. L. Wang X. Rogawski M. A. Weeber E. J. (2017). Effects of the synthetic neurosteroid ganaxolone on seizure activity and behavioral deficits in an Angelman syndrome mouse model. *Neuropharmacology* 116 142–150. 10.1016/j.neuropharm.2016.12.009 27986596

[B23] Colella M. Biran V. Baud O. (2016). Melatonin and the newborn brain. *Early Hum. Dev.* 102 1–3. 10.1016/j.earlhumdev.2016.09.001 27616207

[B24] Committee on Fetus and Newborn Papile L. A. Baley J. E. Benitz W. Cummings J. Carlo W. A. (2014). Hypothermia and neonatal encephalopathy. *Pediatrics* 133 1146–1150. 10.1542/peds.2014-0899 24864176

[B25] Cooper E. J. Johnston G. A. Edwards F. A. (2004). Effects of a naturally occurring neurosteroid on GABAA IPSCs during development in rat hippocampal or cerebellar slices. *J. Physiol.* 521 437–449. 10.1111/j.1469-7793.1999.00437.x PMC2269661 10581314

[B26] Counsell S. Rutherford M. Cowan F. Edwards A. (2003). Magnetic resonance imaging of preterm brain injury. *Arch. Dis. Child. Fetal Neonatal Ed.* 88 F269–F274. 12819156 10.1136/fn.88.4.F269 PMC1721585

[B27] Crestani F. Keist R. Fritschy J. M. Benke D. Vogt K. Prut L. (2002). Trace fear conditioning involves hippocampal alpha5 GABA(A) receptors. *Proc. Natl. Acad. Sci. U.S.A.* 99 8980–8985. 10.1073/pnas.142288699 12084936 PMC124409

[B28] Crossley K. J. Nicol M. B. Hirst J. J. Walker D. W. Thorburn G. D. (1997). Suppression of arousal by progesterone in fetal sheep. *Reprod. Fertil. Dev.* 9 767–773. 9733059 10.1071/r97074

[B29] Crossley K. J. Nitsos I. Walker D. W. Lawrence A. J. Beart P. M. Hirst J. J. (2003). Steroid-sensitive GABAA receptors in the fetal sheep brain. *Neuropharmacology* 45 461–472. 10.1016/s0028-3908(03)00196-5 12907307

[B30] Crossley K. J. Walker D. W. Beart P. M. Hirst J. J. (2000). Characterisation of GABAA receptors in fetal, neonatal and adult ovine brain: region and age related changes and the effects of allopregnanolone. *Neuropharmacology* 39 1514–1522. 10.1016/s0028-3908(99)00222-1 10854896

[B31] Cumberland A. L. Palliser H. K. Crombie G. K. Walker D. W. Hirst J. J. (2017a). Increased anxiety-like phenotype in female guinea pigs following reduced neurosteroid exposure in utero. *Int. J. Dev. Neurosci.* 58 50–58. 10.1016/j.ijdevneu.2017.02.001 28192175

[B32] Cumberland A. L. Palliser H. K. Rani P. Walker D. W. Hirst J. J. (2017b). Effects of combined IUGR and prenatal stress on the development of the hippocampus in a fetal guinea pig model. *J. Dev. Orig. Health. Dis.* 8 584–596. 10.1017/S2040174417000307 28502262

[B33] Cumberland A. L. Palliser H. K. Walker D. W. Hirst J. J. (2017c). Cerebellar changes in guinea pig offspring following suppression of neurosteroid synthesis during late gestation. *Cerebellum* 16 306–313. 10.1007/s12311-016-0802-0 27255705

[B34] Darlow B. Horwood L. Wynn-Williams M. Mogridge N. Austin N. (2009). Admissions of all gestations to a regional neonatal unit versus controls; 2 year outcome. *J. Paediatr. Child Health* 45 187–193. 10.1111/j.1440-1754.2008.01457.x 19320805

[B35] Delaney A. J. Sah P. (1999). GABA receptors inhibited by benzodiazepines mediate fast inhibitory transmission in the central amygdala. *J. Neurosci.* 19 9698–9704. 10.1523/jneurosci.19-22-09698.1999 10559379 PMC6782952

[B36] Djebaili M. Guo Q. Pettus E. Hoffman S. Stein D. (2005). The neurosteroids progesterone and allopregnanolone reduce cell death, gliosis, and funcitonal deficits after traumatic brain injury in rats. *J. Neurotrauma* 22 106–118. 10.1089/neu.2005.22.106 15665606

[B37] Doyle L. W. Crowther C. A. Middleton P. Marret S. Rouse D. (2009). Magnesium sulphate for women at risk of preterm birth for neuroprotection of the fetus. *Cochrane Database Syst. Rev.* 1:CD004661. 10.1089/neu.2005.22.106 19160238

[B38] Dutta R. Chang A. Doud M. K. Kidd G. J. Ribaudo M. V. Young E. A. (2011). Demyelination causes synaptic alterations in hippocampi from multiple sclerosis patients. *Ann. Neurol.* 69 445–454. 10.1002/ana.22337 21446020 PMC3073544

[B39] Elgen S. Sommerfelt K. Leversen K. Markestad T. (2014). Minor neurodevelopmental impairments are associated with increased occurrence of ADHD symptoms in children born extremely preterm. *Eur. Child Adolesc. Psychiatry* 24 463–470. 10.1007/s00787-014-0597-9 25304291

[B40] Essrich C. Lorez M. Benson J. A. Fritschy J. M. Luscher B. (1998). Postsynaptic clustering of major GABAA receptor subtypes requires the gamma 2 subunit and gephyrin. *Nat. Neurosci.* 1 563–571. 10.1038/2798 10196563

[B41] Frey H. A. Klebanoff M. A. (2016). The epidemiology, etiology, and costs of preterm birth. *Semin. Fetal Neonatal Med.* 21 68–73. 10.1016/j.siny.2015.12.011 26794420

[B42] Frye C. A. Walf A. A. (2002). Changes in progesterone metabolites in the hippocampus can modulate open field and forced swim test behavior of proestrous rats. *Horm. Behav.* 41 306–315. 10.1006/hbeh.2002.1763 11971664

[B43] Galinsky R. Draghi V. Wassink G. Davidson J. O. Drury P. P. Lear C. A. (2017). Magnesium sulfate reduces EEG activity but is not neuroprotective after asphyxia in preterm fetal sheep. *J. Cereb. Blood Flow Metab.* 37 1362–1373. 10.1177/0271678X16655548 27317658 PMC5453457

[B44] Ghoumari A. M. Baulieu E. E. Schumacher M. (2005). Progesterone increases oligodendroglial cell proliferation in rat cerebellar slice cultures. *Neuroscience* 135 47–58. 10.1016/j.neuroscience.2005.05.023 16054770

[B45] Ghoumari A. M. Ibanez C. El-Etr M. Leclerc P. Eychenne B. O’Malley B. W. (2003). Progesterone and its metabolites increase myelin basic protein expression in organotypic slice cultures of rat cerebellum. *J. Neurochem.* 86 848–859. 10.1046/j.1471-4159.2003.01881.x 12887683

[B46] Glykys J. Mody I. (2006). Hippocampal network hyperactivity after selective reduction of tonic inhibition in GABA A receptor alpha5 subunit-deficient mice. *J. Neurophysiol.* 95 2796–2807. 10.1152/jn.01122.2005 16452257

[B47] Goldenberg R. L. Culhane J. Iams J. Romero R. (2008). Epidemiology and causes of preterm birth. *Lancet* 371 75–84. 18177778 10.1016/S0140-6736(08)60074-4 PMC7134569

[B48] Gurka M. Locasale-crouch J. Blackman J. (2010). Long-term cognition, acheivment, socioemotional, and behavioural development of health late-preterm infants. *Arch. Pediatr. Adolesc. Med.* 164 525–532. 10.1001/archpediatrics.2010.83 20530302 PMC3287072

[B49] Harney S. C. Frenguelli B. G. Lambert J. J. (2003). Phosphorylation influences neurosteroid modulation of synaptic GABA_A_ receptors in rat CA1 and dentate gyrus neurones. *Neuropharmacology* 45 873–883. 10.1016/s0028-3908(03)00251-x 14529725

[B50] Harrison N. L. Majewska M. D. Harrington J. W. Barker J. L. (1987). Structure-activity relationships for steroid interaction with the gamma-aminobutyric acidA receptor complex. *J. Pharmacol. Exp. Ther.* 241 346–353. 3033209

[B51] Harrison N. L. Simmonds M. A. (1984). Modulation of the GABA receptor complex by a steroid anaesthetic. *Brain Res.* 323 287–292. 10.1016/0006-8993(84)90299-3 6098342

[B52] He J. Evans C. Hoffman S. Oyesiku N. Stein D. (2004). Progesterone and allopregnanolone reduce inflammatory cytokines after traumatic brain injury. *Exp. Neurol.* 189 404–412. 10.1016/j.expneurol.2004.06.008 15380490

[B53] He J. Hoffman S. Stein D. (2003). Allopregnanolone, a progesterone metabolite, enhances behavioural recovery and decreases neuronal loss after traumatic brain injury. *Restor. Neurol. Neurosci.* 22 19–31. 15096691

[B54] Herd M. B. Belelli D. Lambert J. J. (2007). Neurosteroid modulation of synaptic and extrasynaptic GABA(A) receptors. *Pharmacol. Ther.* 116 20–34. 10.1016/j.pharmthera.2007.03.007 17531325

[B55] Herrera T. I. Edwards L. Malcolm W. F. Smith P. B. Fisher K. A. Pizoli C. (2018). Outcomes of preterm infants treated with hypothermia for hypoxic-ischemic encephalopathy. *Early Hum. Dev.* 125 1–7. 10.1016/j.earlhumdev.2018.08.003 30144709

[B56] Hirst J. J. Palliser H. K. Yates D. M. Yawno T. Walker D. W. (2008). Neurosteroids in the fetus and neonate: potential protective role in compromised pregnancies. *Neurochem. Int.* 52 602–610. 10.1016/j.neuint.2007.07.018 17850922

[B57] Hodel A. S. Hunt R. H. Cowell R. A. Van Den Heuvel S. E. Gunnar M. R. Thomas K. M. (2015). Duration of early adversity and structural brain development in post-institutionalized adolescents. *Neuroimage* 105 112–119. 10.1016/j.neuroimage.2014.10.020 25451478 PMC4262668

[B58] Hosie A. M. Wilkins M. E. Smart T. G. (2007). Neurosteroid binding sites on GABA_A_ receptors. *Pharmacol. Ther.* 116 7–19. 17560657 10.1016/j.pharmthera.2007.03.011

[B59] Huddy C. Johnson A. Hope P. (2001). Educational and behavioural problems in babies of 32-35 weeks gestation. *Arch. Dis. Child.* 85 F23–F28. 11420317 10.1136/fn.85.1.F23 PMC1721280

[B60] Jacobs S. E. Berg M. Hunt R. Tarnow-Mordi W. O. Inder T. E. Davis P. G. (2013). Cooling for newborns with hypoxic ischaemic encephalopathy. *Cochrane Database Syst. Rev.* 1:CD003311. 10.1002/14651858.CD003311 14583966

[B61] Kajantie E. Phillips D. I. Andersson S. Barker D. J. Dunkel L. Forsén T. (2002). Size at birth, gestational age and cortisol secretion in adult life: foetal programming of both hyper- and hypocortisolism? *Clin. Endocrinol.* 57 635–641. 10.1046/j.1365-2265.2002.01659.x 12390338

[B62] Kamyar M. Manuck T. A. Stoddard G. J. Varner M. W. Clark E. (2016). Magnesium sulfate, chorioamnionitis, and neurodevelopment after preterm birth. *BJOG* 123 1161–1166. 10.1111/1471-0528.13460 26036660

[B63] Kapoor A. Dunn E. Kostaki A. Andrews M. H. Matthews S. G. (2006). Fetal programming of hypothalamo-pituitary-adrenal function: prenatal stress and glucocorticoids. *J. Physiol.* 572 31–44. 10.1113/jphysiol.2006.105254 16469780 PMC1779638

[B64] Kapoor A. Matthews S. G. (2005). Short periods of prenatal stress affect growth, behaviour and hypothalamo-pituitary-adrenal axis activity in male guinea pig offspring. *J. Physiol.* 566 967–977. 10.1113/jphysiol.2005.090191 15932885 PMC1464791

[B65] Kapoor A. Matthews S. G. (2008). Prenatal stress modifies behavior and hypothalamic-pituitary-adrenal function in female guinea pig offspring: effects of timing of prenatal stress and stage of reproductive cycle. *Endocrinology* 149 6406–6415. 10.1210/en.2008-0347 18755800

[B66] Kazdoba T. M. Hagerman R. J. Zolkowska D. Rogawski M. A. Crawley J. N. (2016). Evaluation of the neuroactive steroid ganaxolone on social and repetitive behaviors in the BTBR mouse model of autism. *Psychopharmacology* 233 309–323. 10.1007/s00213-015-4115-7 26525567 PMC4703522

[B67] Kelleher M. A. Hirst J. J. Palliser H. K. (2013). Changes in neuroactive steroid concentrations after preterm delivery in the Guinea pig. *Reprod. Sci.* 20 1365–1375. 10.1177/1933719113485295 23585339 PMC3795424

[B68] Kelleher M. A. Palliser H. K. Hirst J. J. (2011a). “Neurosteroid replacement therapy in the preterm neonate,” in *Proceedings of the 38th annual meeting of the Fetal and Neonatal Physiological Society*, (Palm Cove, QLD).

[B69] Kelleher M. A. Palliser H. K. Walker D. W. Hirst J. J. (2011b). Sex-dependent effect of a low neurosteroid environment and intrauterine growth restriction on foetal guinea pig brain development. *J. Endocrinol.* 208 301–309. 10.1677/JOE-10-0248 21149437

[B70] Kim P. Choi C. S. Park J. H. Joo S. H. Kim S. Y. Ko H. M. (2014). Chronic exposure to ethanol of male mice before mating produces attention deficit hyperactivity disorder-like phenotype along with epigenetic dysregulation of dopamine transporter expression in mouse offspring. *J. Neurosci. Res.* 92 658–670. 10.1002/jnr.23275 24510599

[B71] Kirkegaard I. Obel C. Hedegaard M. Henriksen T. (2006). Gestational age and birth weight in relation to school performance of 10 year old children: a follow-up study of childrenborn after 32 completed weeks. *Pediatrics* 118 1600–1606. 10.1542/peds.2005-2700 17015552

[B72] Koning G. Leverin A. L. Nair S. Schwendimann L. Ek J. Carlsson Y. (2017). Magnesium induces preconditioning of the neonatal brain via profound mitochondrial protection. *J. Cereb. Blood Flow Metab.* [Epub ahead of print], 29206066 10.1177/0271678X17746132 PMC6547197

[B73] Lambert J. Peters J. Cottrell G. (1987). Actions of synthetic and endogenous steroids on the GABA_A_ receptor. *Trends Pharmacol. Sci.* 8 224–227.

[B74] Lambert J. J. Belelli D. Peden D. R. Vardy A. W. Peters J. A. (2003). Neurosteroid modulation of GABA_A_ receptors. *Prog. Neurobiol.* 71 67–80. 14611869 10.1016/j.pneurobio.2003.09.001

[B75] Laptook A. R. (2017). Therapeutic hypothermia for preterm infants with hypoxic-ischemic encephalopathy: how do we move forward? *J. Pediatr.* 183 8–9. 10.1016/j.jpeds.2016.12.074 28108104

[B76] Lee H. H. Deeb T. Z. Walker J. A. Davies P. A. Moss S. J. (2011). NMDA receptor activity downregulates KCC2 resulting in depolarizing GABAA receptor-mediated currents. *Nat. Neurosci.* 14 736–743. 10.1038/nn.2806 21532577 PMC3102766

[B77] Lee H. H. Walker J. A. Williams J. R. Goodier R. J. Payne J. A. Moss S. J. (2007). Direct protein kinase C-dependent phosphorylation regulates the cell surface stability and activity of the potassium chloride cotransporter KCC2. *J. Biol. Chem.* 282 29777–29784. 10.1074/jbc.m705053200 17693402

[B78] Lekic T. Manaenko A. Rolland W. Virbel K. Hartman R. Tang J. (2011). Neuroprotection by melatonin after germinal matrix hemorrhage in neonatal rats. *Acta Neurochir. Suppl.* 111 201–206. 10.1007/978-3-7091-0693-8_34 21725756 PMC3569074

[B79] Liao G. Cheung S. Galeano J. Ji A. X. Qin Q. Bi X. (2009). Allopregnanolone treatment delays cholesterol accumulation and reduces autophagic/lysosomal dysfunction and inflammation in Npc1-/- mouse brain. *Brain Res.* 1270 140–151. 10.1016/j.brainres.2009.03.027 19328188 PMC2677133

[B80] Lindahl J. Kjellsen B. Tigert J. Miskimins R. (2008). In utero PCP exposure alters oligodendrocyte differentiation and myelination in developing rat frontal cortex. *Brain Res.* 1234 137–147. 10.1016/j.brainres.2008.06.126 18675260 PMC2572227

[B81] Lindstrom K. Lindblad F. Hjern A. (2011). Preterm birth and attention-deficit hyperactivity disorder in schoolchildren. *Pediatrics* 127 858–865. 10.1542/peds.2010-1279 21502231

[B82] Linnet K. Wisborg K. Agerbo E. Secher N. Thomsen P. Henriksen T. (2006). Gestational age, birth weight, and the risk of hyperkinetic disorder. *Arch. Dis. Child* 91 655–660. 10.1136/adc.2005.088872 16754656 PMC2083047

[B83] Loe I. M. Lee E. S. Luna B. Feldman H. M. (2011). Behavior problems of 9-16 year old preterm children: biological, sociodemographic, and intellectual contributions. *Early Hum. Dev.* 87 247–252. 10.1016/j.earlhumdev.2011.01.023 21316875 PMC3180905

[B84] Lombardi I. Luisi S. Quirici B. Monteleone P. Bernardi F. Liut M. (2004). Adrenal response to adrenocorticotropic hormone stimulation in patients with premenstrual syndrome. *Gynecol. Endocrinol.* 18 79–87. 10.1080/09513590310001652955 15195499

[B85] Loria C. J. Stevens A. M. Crummy E. Casadesus G. Jacono F. J. Dick T. E. (2013). Respiratory and behavioral dysfunction following loss of the GABAA receptor alpha4 subunit. *Brain Behav.* 3 104–113. 10.1002/brb3.122 23533098 PMC3607152

[B86] Luchetti S. Huitinga I. Swaab D. F. (2011). Neurosteroid and GABA-A receptor alterations in Alzheimer’s disease, Parkinson’s disease and multiple sclerosis. *Neuroscience* 191 6–21. 10.1016/j.neuroscience.2011.04.010 21514366

[B87] Macdonald R. L. Botzolakis E. (2009). “GABAA receptor channels,” in *Physiology and Pathology of Chloride Transporters and Channels in the Nervous System: From Molecules to Diseases*, eds Javier Alvarez-Leefmans F. Delpire E. (Amsterdam: Elsevier Science).

[B88] Majewska M. D. (1992). Neurosteroids: endogenous bimodal modulators of the GABAA receptor. *Mechanism of action and physiological significance*. *Prog. Neurobiol.* 38 379–395. 1349441 10.1016/0301-0082(92)90025-a

[B89] Marret S. Ancel P. Marpeau L. (2007). Neonatal and 5 year outcomes after birth at 30-34 weeks gestation. *Obstet Gynecol* 110 72–80. 10.1097/01.aog.0000267498.95402.bd 17601899

[B90] Martini L. Celotti F. Melcangi R. (1996). Testosterone and progesterone metabolism in the central nervous system: cellular localization and mechanism of control of the enzymes involved. *Cell. Mol. Neurobiol.* 16 271–282. 10.1007/bf02088095 8818396 PMC11563072

[B91] Mathews T. Menacker F. MacDorman M. F. (2002). Infant mortality statistics from the period linked birth/infant death data set. *Natl. Vital Stat. Rep.* 2004 1–32. 15622996

[B92] Matsusue Y. Horii-Hayashi N. Kirita T. Nishi M. (2014). Distribution of corticosteroid receptors in mature oligodendrocytes and oligodendrocyte progenitors of the adult mouse brain. *J. Histochem. Cytochem.* 62 211–226. 10.1369/0022155413517700 24309510 PMC3935446

[B93] McKendry A. Palliser H. Yates D. Walker D. Hirst J. (2009). The effect of betamethasone treatment on neuroactive steroid synthesis in a foetal guinea pig model of growth restriction. *J. Neuroendocrinol.* 22 166–174. 10.1111/j.1365-2826.2009.01949.x 20041984

[B94] Mellon S. H. Gong W. Schonemann M. D. (2008). Endogenous and synthetic neurosteroids in treatment of Niemann-Pick Type C disease. *Brain Res. Rev.* 57 410–420. 10.1016/j.brainresrev.2007.05.012 17629950 PMC2323675

[B95] Mellon S. H. Griffin L. D. (2002). Neurosteroids: biochemistry and clinical significance. *Trends Endocrinol. Metab.* 13 35–43. 10.1016/s1043-2760(01)00503-3 11750861

[B96] Mihalek R. M. Banerjee P. K. Korpi E. R. Korpi E. R. Quinlan J. J. Firestone L. L. (1999). Attenuated sensitivity to neuroactive steroids in γ-aminobutyrate type A receptor delta subunit knockout mice. *Proc. Natl. Acad. Sci. U.S.A.* 96 12905–12910. 10.1073/pnas.96.22.12905 10536021 PMC23157

[B97] Mirmiran M. (1995). The function of fetal/neonatal rapid eye movement sleep. *Behav. Brain Res.* 69 13–22. 10.1016/0166-4328(95)00019-p 7546304

[B98] Monaghan E. P. Navalta L. A. Shum L. Ashbrook D. W. Lee D. A. (1997). Initial human experience with ganaxolone, a neuroactive steroid with antiepileptic activity. *Epilepsia* 38 1026–1031. 10.1111/j.1528-1157.1997.tb01486.x 9579942

[B99] Monteleone P. Luisi S. Tonetti A. Bernardi F. Genazzani A. D. Luisi M. (2000). Allopregnanolone concentrations and premenstrual syndrome. *Eur. J. Endocrinol.* 142 269–273. 10.1530/eje.0.1420269 10700721

[B100] Morrison J. L. Botting K. J. Darby J. R. T. Dyson R. M. Gatford K. L. Gray C. (2018). Invited review: guinea pig models for translation of DOHAD into the clinic. *J. Physiol.* 596 5535–5569. 10.1113/JP274948 29633280 PMC6265540

[B101] Morse S. Zheng H. Tang Y. Roth J. (2009). Early school-age outcomes of late preterm infants. *Pediatrics* 123 622–629. 10.1542/peds.2008-1405 19336353

[B102] Moster D. Lie R. T. Markestad T. (2008). Long-term medical and social consequences of preterm birth. *N. Engl. J. Med.* 359 262–273. 10.1056/NEJMoa0706475 18635431

[B103] Nguyen P. N. Billiards S. S. Walker D. W. Hirst J. J. (2003). Changes in 5 alpha-pregnane steroids and neurosteroidogenic enzyme expression in fetal sheep with umbilicoplacental embolization. *Pediatr. Res.* 54 840–847. 10.1203/01.pdr.0000088066.47755.36 12930920

[B104] Nguyen P. N. Yan E. B. Castillo-Melendez M. Walker D. W. Hirst J. J. (2004). Increased allopregnanolone levels in the fetal sheep brain following umbilical cord occlusion. *J. Physiol.* 560 593–602. 10.1113/jphysiol.2004.069336 15331682 PMC1665267

[B105] Nicol M. Hirst J. Walker D. (1998). Effect of pregnane steroids on electrocortical activity and somatosensory evoked potentials in fetal sheep. *Neurosci. Lett.* 253 111–114. 10.1016/s0304-3940(98)00627-2 9774162

[B106] Nicol M. Hirst J. Walker D. Thorburn G. (1997). Effect of alteration of maternal plasma progesterone concentrations on fetal behavioural state during late gestation. *J. Endocrinol.* 152 379–386. 10.1677/joe.0.1520379 9071958

[B107] Nicol M. B. Hirst J. J. Walker D. W. (2001). Effect of finasteride on behavioural arousal and somatosensory evoked potentials in fetal sheep. *Neurosci. Lett.* 306 13–16. 10.1016/s0304-3940(01)01861-4 11403946

[B108] Nohria V. Giller E. (2007). Ganaxolone. *Neurotherapeutics* 4 102–105. 10.1016/j.nurt.2006.11.003 17199022 PMC7479704

[B109] Olsen R. Sapp D. (1995). Neuroactive steroid modulation of GABAA receptors. *Adv. Biochem. Psychopharmacol.* 48 57–74. 7653326

[B110] Owens D. F. Kriegstein A. R. (2002). Is there more to GABA than synaptic inhibition? *Nat. Rev. Neurosci.* 3 715–727. 10.1038/nrn919 12209120

[B111] Palliser H. K. Kelleher M. A. Tolcos M. Walker D. W. Hirst J. J. (2015). Effect of postnatal progesterone therapy following preterm birth on neurosteroid concentrations and cerebellar myelination in guinea pigs. *J. Dev. Orig. Health. Dis.* 6 350–361. 10.1017/S2040174415001075 25907069

[B112] Paris J. J. Brunton P. J. Russell J. A. Walf A. A. Frye C. A. (2011). Inhibition of 5alpha-reductase activity in late pregnancy decreases gestational length and fecundity and impairs object memory and central progestogen milieu of juvenile rat offspring. *J. Neuroendocrinol.* 23 1079–1090. 10.1111/j.1365-2826.2011.02219.x 21914008 PMC3196810

[B113] Paul S. M. Purdy R. (1992). Neuroactive steroids. *FASEB J.* 6 2311–2322. 1347506

[B114] Petrini J. Dias T. McCormick M. Massolo M. Green N. Escobar G. (2009). Increased risk of adverse neurological development for late preterm infants. *J. Pediatr.* 154 169–176. 10.1016/j.jpeds.2008.08.020 19081113

[B115] Pinna G. Rasmusson A. M. (2014). Ganaxolone improves behavioral deficits in a mouse model of post-traumatic stress disorder. *Front. Cell Neurosci.* 8:256. 10.3389/fncel.2014.00256 25309317 PMC4161165

[B116] Potijk M. R. de Winter A. F. Bos A. F. Kerstjens J. M. Reijneveld S. A. (2012). Higher rates of behavioural and emotional problems at preschool age in children born moderately preterm. *Arch. Dis. Child.* 97 112–117. 10.1136/adc.2011.300131 22147746

[B117] Rao R. Trivedi S. Vesoulis Z. Liao S. M. Smyser C. D. Mathur A. M. (2017). Safety and short-term outcomes of therapeutic hypothermia in preterm neonates 34-35 weeks gestational age with hypoxic-ischemic encephalopathy. *J. Pediatr.* 183 37–42. 10.1016/j.jpeds.2016.11.019 27979578 PMC5367984

[B118] Rasmusson A. M. Pinna G. Paliwal P. Weisman D. Gottschalk C. Charney D. (2006). Decreased cerebrospinal fluid allopregnanolone levels in women with posttraumatic stress disorder. *Biol. Psychiatry* 60 704–713. 10.1016/j.biopsych.2006.03.026 16934764

[B119] Reddy D. S. Rogawski M. A. (2012). “Neurosteroids - endogenous regulators of seizure susceptibility and role in the treatment of epilepsy,” in *Jasper’s Basic Mechanisms of the Epilepsies*, 4th Edn, eds Noebels J. L. Avoli M. Rogawski M. A. Olsen R. W. Delgado-Escueta A. V. (Rockville, MD: Bethesda). 22787590

[B120] Rees S. Harding R. Walker D. (2008). An adverse intrauterine environment: implications for injury and altered development of the brain. *Int. J. Dev. Neurosci.* 26 3–11. 10.1016/j.ijdevneu.2007.08.020 17981423

[B121] Rees S. Inder T. (2005). Fetal and neonatal origins of altered brain development. *Early Hum. Dev.* 81 753–761. 10.1016/j.earlhumdev.2005.07.004 16107304

[B122] Represa A. Ben-Ari Y. (2005). Trophic actions of GABA on neuronal development. *Trends Neurosci.* 28 278–283. 10.1016/j.tins.2005.03.010 15927682

[B123] Rivera C. Voipio J. Kaila K. (2005). Two developmental switches in GABAergic signalling: the K+-Cl- cotransporter KCC2 and carbonic anhydrase CAVII. *J. Physiol.* 562 27–36. 10.1113/jphysiol.2004.077495 15528236 PMC1665491

[B124] Rivera C. Voipio J. Payne J. A. Ruusuvuori E. Lahtinen H. Lamsa K. (1999). The K+/Cl- co-transporter KCC2 renders GABA hyperpolarizing during neuronal maturation. *Nature* 397 251–255. 10.1038/16697 9930699

[B125] Rivkin M. J. (1997). Hypoxic-ischemic brain injury in the term newborn. Neuropathology, clinical aspects, and neuroimaging. *Clin. Perinatol.* 24 607–625. 10.1016/s0095-5108(18)30161-1 9394863

[B126] Romeo D. Di Stefano A. Conversano M. (2010). Neurodevelopmental outcome at 12 and 18 months in late preterm infants. *Eur. J. Paediatr. Neurol.* 14 503–507. 10.1016/j.ejpn.2010.02.002 20207178

[B127] Roof R. Duvdevani R. Braswell L. Stein D. (1994). Progesterone facilitates cognitive recovery and reduces secondary neuronal loss caused by cortical contusion injury in male rats. *Exp. Neurol.* 129 64–69. 10.1006/exnr.1994.1147 7925843

[B128] Roof R. Duvdevani R. Heyburn J. Stein D. (1996). Progesterone rapidly decreases brain edema: treatment delayed up to 24 hours is still effective. *Exp. Neurol.* 138 246–251. 10.1006/exnr.1996.0063 8620923

[B129] Schendel D. Bhasin T. (2008). Birth weight and gestational age characteristics of children with autism, including a comparison with other developmental disabilities. *Pediatrics* 121 1155–1164. 10.1542/peds.2007-1049 18519485

[B130] Schermann L. Sedin G. (2004). Cognitive function at 10 years of age in children who have required neonatal intensive care. *Acta Paediatr.* 93 1619–1629. 10.1111/j.1651-2227.2004.tb00853.x 15841771

[B131] Schumacher M. Guennoun R. Robert F. Carelli C. Gago N. Ghoumari A. (2004). Local synthesis and dual actions of progesterone in the nervous system: neuroprotection and myelination. *Growth Horm. IGF Res.* 14(Suppl. A), S18–S33. 15135772 10.1016/j.ghir.2004.03.007

[B132] Shaw J. C. Dyson R. M. Palliser H. K. Gray C. Berry M. J. Hirst J. J. (2018). Neurosteroid replacement therapy using the allopregnanolone-analogue ganaxolone following preterm birth in male guinea pigs. *Pediatr. Res.* 85 86–96. 10.1038/s41390-018-0185-7 30237570

[B133] Shaw J. C. Palliser H. K. Dyson R. M. Berry M. J. Hirst J. J. (2017). Disruptions to the cerebellar GABAergic system in juvenile guinea pigs following preterm birth. *Int. J. Dev. Neurosci.* 65 1–10. 10.1016/j.ijdevneu.2017.10.002 29024720

[B134] Shaw J. C. Palliser H. K. Dyson R. M. Hirst J. J. Berry M. J. (2016). Long-term effects of preterm birth on behavior and neurosteroid sensitivity in the guinea pig. *Pediatr. Res.* 80 275–283. 10.1038/pr.2016.63 27055188

[B135] Shaw J. C. Palliser H. K. Walker D. W. Hirst J. (2015). Preterm birth affects GABAA receptor subunit mRNA levels during the foetal-to-neonatal transition in guinea pigs. *J. Dev. Orig. Health Dis.* 6 250–260. 10.1017/S2040174415000069 25661827

[B136] Sieghart W. Fuchs K. Tretter V. Ebert V. Jechlinger M. Höger H. (1999). Structure and subunit composition of GABA_A_ receptors. *Neurochem. Int.* 34 379–385. 10397365 10.1016/s0197-0186(99)00045-5

[B137] Simmons L. E. Rubens C. E. Darmstadt G. L. Gravett M. G. (2010). Preventing preterm birth and neonatal mortality: exploring the epidemiology, causes, and interventions. *Semin. Perinatol.* 34 408–415. 10.1053/j.semperi.2010.09.005 21094415

[B138] Singh G. Kenney M. Ghandour R. Kogan M. Lu M. (2013). Mental health outcomes in US children and adolescents born prematurely or with low birthwight. *Dep. Res. Treat.* 2013:570743. 10.1155/2013/570743 24324882 PMC3845867

[B139] Sperling M. R. Klein P. Tsai J. (2017). Randomized, double-blind, placebo-controlled phase 2 study of ganaxolone as add-on therapy in adults with uncontrolled partial-onset seizures. *Epilepsia* 58 558–564. 10.1111/epi.13705 28230252

[B140] Spigelman I. Li Z. Banerjee P. K. Mihalek R. M. Homanics G. E. Olsen R. W. (2002). Behavior and physiology of mice lacking the GABAA-receptor delta subunit. *Epilepsia* 43(Suppl. 5), 3–8. 10.1046/j.1528-1157.43.s.5.8.x 12121286

[B141] Spigelman I. Li Z. Liang J. Samzadeh S. Mihalek R. M. Homanics G. E. (2003). Reduced inhibition and sensitivity to neurosteroids in hippocampus of mice lacking the GABA(A) receptor delta subunit. *J. Neurophysiol.* 90 903–910. 10.1152/jn.01022.2002 12702713

[B142] Stell B. M. Brickley S. G. Tang C. Farrant M. (2003). Neuroactive steroids reduce neuronal excitability by selectively enhancing tonic inhibition mediated by δ subunit-containing GABAA receptors. *Proc. Natl. Acad. Sci.* 100 14439–14444. 10.1073/pnas.2435457100 14623958 PMC283610

[B143] Stoffel-Wagner B. (2001). Neurosteroid metabolism in the human brain. *Eur. J. Endocrinol.* 145 669–679. 10.1530/eje.0.1450669 11720889

[B144] Stoodley C. J. (2012). The cerebellum and cognition: evidence from functional imaging studies. *Cerebellum* 11 352–365. 10.1007/s12311-011-0260-7 21373864

[B145] Stoodley C. J. Schmahmann J. D. (2010). Evidence for topographic organization in the cerebellum of motor control versus cognitive and affective processing. *Cortex* 46 831–844. 10.1016/j.cortex.2009.11.008 20152963 PMC2873095

[B146] Strohle A. Romeo E. Hermann B. Pasini A. Spalletta G. di Michele F. (1999). Concentrations of 3 alpha-reduced neuroactive steroids and their precursors in plasma of patients with major depression and after clinical recovery. *Biol. Psychiatry* 45 274–277. 10.1016/s0006-3223(98)00328-x 10023501

[B147] Talge N. Holzman C. Wang J. Lucia V. Gardiner J. Breslau N. (2010). Late-preterm birth and its association with cognitive and socioemotional outcomes at 6 years of age. *Pediatrics* 126 1124–1131. 10.1542/peds.2010-1536 21098151

[B148] van Baar A. L. Vermaas J. Knots E. de Kleine M. J. Soons P. (2009). Functioning at school age of moderately preterm children born at 32 to 36 weeks’ gestational age. *Pediatrics* 124 251–257. 10.1542/peds.2008-2315 19564307

[B149] van Tilborg E. Heijnen C. J. Benders M. J. van Bel F. Fleiss B. Gressens P. (2016). Impaired oligodendrocyte maturation in preterm infants: potential therapeutic targets. *Prog. Neurobiol.* 136 28–49. 10.1016/j.pneurobio.2015.11.002 26655283

[B150] Verkuyl J. M. Hemby S. E. Joels M. (2004). Chronic stress attenuates GABAergic inhibition and alters gene expression of parvocellular neurons in rat hypothalamus. *Eur. J. Neurosci.* 20 1665–1673. 10.1111/j.1460-9568.2004.03568.x 15355334

[B151] Vicari S. Caravale B. Carlesimo G. A. Casadei A. M. Allemand F. (2004). Spatial working memory deficits in children at ages 3-4 who were low birth weight, preterm infants. *Neuropsychology* 18 673–678. 10.1037/0894-4105.18.4.673 15506835

[B152] Villapol S. Fau S. Renolleau S. Biran V. Charriaut-Marlangue C. Baud O. (2011). Melatonin promotes myelination by decreasing white matter inflammation after neonatal stroke. *Pediatr. Res.* 69 51–55. 10.1203/PDR.0b013e3181fcb40b 20856166

[B153] Vollmer B. Lundequist A. Martensson G. Nagy Z. Lagercrantz H. Smedler A. C. (2017). Correlation between white matter microstructure and executive functions suggests early developmental influence on long fibre tracts in preterm born adolescents. *PLoS One* 12:e0178893. 10.1371/journal.pone.0178893 28594884 PMC5464584

[B154] Volpe J. (2009). Brain injury in premature infants: a complex amalgam of destructive and developmental disturbances. *Lancet Neurol.* 8 110–124. 10.1016/S1474-4422(08)70294-1 19081519 PMC2707149

[B155] Volpe J. J. (2001). Neurobiology of periventricular leukomalacia in the premature infant. *Pediatr. Res.* 50 553–562. 10.1203/00006450-200111000-00003 11641446

[B156] Volpe J. J. (2003). Cerebral white matter injury of the premature infant-more common than you think. *Pediatrics* 112 176–180. 10.1542/peds.112.1.176 12837883

[B157] Volpe J. J. (2008). *Neurology of the Newborn.* Amsterdam: Elsevier Health Sciences.

[B158] Walf A. A. Sumida K. Frye C. A. (2006). Inhibiting 5alpha-reductase in the amygdala attenuates antianxiety and antidepressive behavior of naturally receptive and hormone-primed ovariectomized rats. *Psychopharmacology* 186 302–311. 10.1007/s00213-005-0100-x 16220340 PMC3608208

[B159] Wilkinson D. Shepherd E. Wallace E. M. (2016). Melatonin for women in pregnancy for neuroprotection of the fetus. *Cochrane Database Syst. Rev.* 3:CD010527. 10.1002/14651858.CD010527.pub2 27022888 PMC7081745

[B160] Williamson A. Mellor J. Grant A. Randall A. (1998). Properties of GABA_A_ receptors in cultured rat oligodendrocyte progenitor cells. *Neuropharmacology* 37 859–873. 10.1016/s0028-3908(98)00016-1 9776382

[B161] Woythaler M. McCormick M. Smith V. (2011). Late preterm infants have worse 24 month neurodevelopmental outcomes than term infants. *Pediatrics* 127 622–629. 10.1542/peds.2009-3598 21321024

[B162] Wright D. W. Kellermann A. L. Hertzberg V. S. Clark P. L. Frankel M. Goldstein F. C. (2007). ProTECT: a randomized clinical trial of progesterone for acute traumatic brain injury. *Ann. Emerg. Med.* 49 391–402. 17011666 10.1016/j.annemergmed.2006.07.932

[B163] Xiao G. Wei J. Yan W. Wang W. Lu Z. (2008). Improved outcomes from the administration of progesterone for patients with acute severe traumatic brain injury: a randomized controlled trial. *Crit. Care* 12:R61. 10.1186/cc6887 18447940 PMC2447617

[B164] Yawno T. Hirst J. J. Castillo-Melendez M. Walker D. W. (2009). Role of neurosteroids in regulating cell death and proliferation in the late gestation fetal brain. *Neuroscience* 163 838–847. 10.1016/j.neuroscience.2009.07.009 19591903

[B165] Yawno T. Miller S. L. Bennet L. Wong F. Hirst J. J. Fahey M. (2017). Ganaxolone: a new treatment for neonatal seizures. *Front. Cell Neurosci.* 11:246. 10.3389/fncel.2017.00246 28878622 PMC5572234

[B166] Yawno T. Yan E. Walker D. Hirst J. (2007). Inhibition of neurosteroid synthesis increases asphyxia-induced brain injury in the late gestation fetal sheep. *Neuroscience* 146 1726–1733. 10.1016/j.neuroscience.2007.03.023 17449186

[B167] Yawno T. Yan E. B. Hirst J. J. Walker D. W. (2011). Neuroactive steroids induce changes in fetal sheep behavior during normoxic and asphyxic states. *Stress* 14 13–22. 10.3109/10253890.2010.504789 20828337

[B168] Yu Z. Y. Wang W. Fritschy J. M. Witte O. W. Redecker C. (2006). Changes in neocortical and hippocampal GABAA receptor subunit distribution during brain maturation and aging. *Brain Res.* 1099 73–81. 10.1016/j.brainres.2006.04.118 16781682

[B169] Yum M. S. Lee M. Ko T. S. Velisek L. (2014). A potential effect of ganaxolone in an animal model of infantile spasms. *Epilepsy Res.* 108 1492–1500. 10.1016/j.eplepsyres.2014.08.015 25219352

